# Anti-Inflammatory Activity of Diterpenoids from *Celastrus orbiculatus* in Lipopolysaccharide-Stimulated RAW264.7 Cells

**DOI:** 10.1155/2020/7207354

**Published:** 2020-07-30

**Authors:** Hyun-Jae Jang, Kang-Hoon Kim, Eun-Jae Park, Jeong A. Kang, Bong-Sik Yun, Seung-Jae Lee, Chan Sun Park, Soyoung Lee, Seung Woong Lee, Mun-Chual Rho

**Affiliations:** ^1^Immunoregulatory Materials Research Center, Korea Research Institute of Bioscience and Biotechnology, 181 Ipsin-gil, Jeongeup-si, Jeonbuk 56212, Republic of Korea; ^2^Division of Biotechnology and Advanced Institute of Environment and Bioscience, College of Environmental and Bioresource Sciences, Jeonbuk National University, Iksan-si, Republic of Korea

## Abstract

*Celastrus orbiculatus* Thunb has been known as an ethnopharmacological medicinal plant for antitumor, anti-inflammatory, and analgesic effects. Although various pharmacological studies of *C*. *orbiculatus* extract has been reported, an anti-inflammatory mechanism study of their phytochemical constituents has not been fully elucidated. In this study, compounds **1**–**17**, including undescribed podocarpane-type trinorditerpenoid (**3**), were purified from *C*. *orbiculatus* and their chemical structure were determined by high-resolution electrospray ionization mass (HRESIMS) and nuclear magnetic resonance (NMR) spectroscopic data. To investigate the anti-inflammatory activity of compounds **1**–**17**, nitric oxide (NO) secretion was evaluated in LPS-treated murine macrophages, RAW264.7 cells. Among compounds **1**–**17**, deoxynimbidiol (**1**) and new trinorditerpenoid (**3**) showed the most potent inhibitory effects (IC_50_: 4.9 and 12.6 *μ*M, respectively) on lipopolysaccharide- (LPS-) stimulated NO releases as well as proinflammatory mediators, such as inducible nitric oxide (iNOS), cyclooxygenase- (COX-) 2, interleukin- (IL-) 1*β*, IL-6, and tumor necrosis factor- (TNF-) *α*. Its inhibitory activity of proinflammatory mediators is contributed by suppressing the activation of nuclear transcription factor- (NF-) *κ*B and mitogen-activated protein kinase (MAPK) signaling cascades including p65, inhibition of NF-*κ*B (I*κ*B), extracellular signal-regulated kinase (ERK), c-Jun NH_2_-terminal kinase (JNK), and p38. Therefore, these results demonstrated that diterpenoids **1** and **3** obtained from *C*. *orbiculatus* may be considered a potential candidate for the treatment of inflammatory diseases.

## 1. Introduction

*Celastrus orbiculatus* Thunb. (Oriental bittersweet) is a perennial woody vine belonging to the family Celastraceae, which is native to East Asia including China, Japan, and Korea [1, 2]. *C*. *orbiculatus* has been traditionally prescribed as a herbal remedy for bacterial infection, insecticidal, and rheumatoid arthritis [3, 4]. Previous pharmacological studies has shown that these extracts containing diverse phytochemical components such as sesquiterpenoids, diterpenoids, triterpenoids, alkaloids, flavonoids, and phenolic compounds [5–10] exhibit various biological activity such as antitumor [11–14], antioxidant [9], antinociceptive [15], antiatherosclerosis [16], neuroprotective [17], and anti-inflammatory [18] effects. Although a variety of biological activities of *C*. *orbiculatus* extracts reported in the literatures, whether any phytochemical component contributes to their biological mechanisms other than celastrol, which is the main triterpenoid compound of *C*. *orbiculatus* [19, 20], has been discussed limitedly so far.

The major function of the inflammation is to defend the host from infectious pathogens and repair tissue injury through the action of leukocytes including macrophages, neutrophils, and lymphocytes [21, 22]. However, immoderate or prolonged inflammation contribute to the development of chronic inflammation diseases such as arthritis, asthma, Crohn's, and inflammatory bowel disease (IBD), resulting in swelling, pain, and eventually damage of tissue or organ dysfunction [23, 24]. Macrophage activated by antigen, pathogens, and endogenous inflammatory stimuli is associated with functional and physiological changes in the cells and generates proinflammatory and cytotoxic mediators such as nitric oxide (NO), tumor necrosis factor *α* (TNF-*α*), interleukin-1*β* (IL-1*β*), IL-6, reactive oxygen mediators, and hydrolytic enzymes [25, 26]. Excessive NO and inflammatory cytokines released from macrophages are implicated in cytotoxicity by initiating both apoptosis and necrosis of normal tissues as well as destruction of tumor cells and exogenous pathogens [27, 28]. Thus, blocking these inflammatory mediators is considered to be effective for prevention of inflammation diseases.

Binding of these inflammatory mediators or bacterial lipopolysaccharide (LPS) to specific receptors including Toll-like receptors (TLRs) lead to the inflammatory responses, through the transmembrane signal transduction and intracellular responses such as nuclear transcription factor-*κ*B (NF-*κ*B) and mitogen-activated protein kinases (MAPKs) [29, 30]. The activation of NF-*κ*B is involved in the phosphorylation of I*κ*B, resulting in the release of NF-*κ*B into the nucleus, which functions as a transcription factor for expressing proinflammatory target genes including inducible nitric oxide synthesis (iNOS), cyclooxygenase 2 (COX-2), TNF-*α*, IL-1*β*, and IL-6 [31]. Extracellular signal-regulated kinase (ERK), c-Jun NH_2_-terminal kinase (JNK), and p38 kinase are generally known as subfamilies of MAPKs, and this phosphorylation involved in NF-*κ*B activation modulates proinflammation mediators, such as iNOS and COX-2 in activated macrophages [23, 32, 33]. Therefore, the development of natural sources targeting the NF-*κ*B and MAPK cascades can be a potential therapeutic for inflammatory diseases.

In current study, the chemical structures of phytochemical constituents (**1**–**17**) isolated from *C*. *orbiculatus* were determined by spectroscopic data including NMR and ESI-MS. Among components obtained from *C*. *orbiculatus*, compounds **1** and **3**, both of which are podocarpane trinorditerpenoids, exhibited most potent inhibitory activity against LPS-treated NO release, and their anti-inflammatory activity was explored through underlying molecular mechanisms including NF-*κ*B and MAPK signaling pathway.

## 2. Materials and Methods

### 2.1. General Experimental Procedures

Column chromatography was performed with silica gel (Kieselgel 60, 230-400 mesh, Merck, Darmstadt, Germany), and silica gel 60 F_254_ and RP-18 F_254_s (Merck) were used for TLC analysis. Medium-pressure liquid chromatography (MPLC) was performed using a Combiflash RF (Teledyne Isco, Lincoln, NE, USA), and semipreparative HPLC was performed on a Shimadzu LC-6 AD (Shimadzu Co., Tokyo, Japan) instrument equipped with a SPD-20A detector using Phenomenex Luna C_18_ (250 × 21.2 mm, 5 *μ*m, Phenomenex, Torrance, CA, USA), Phenomenex Kinetex C_18_ (150 × 21.2 mm, 5 *μ*m), Phenomenex Luna C_8_ (150 × 21.2 mm, 5 *μ*m), and YMC C_18_ J'sphere ODS H80 (250 × 20 mm, 4 *μ*m, YMC Co., Kyoto, Japan) columns. ^1^H-, ^13^C-, and 2D NMR spectroscopic data were measured on a JEOL JNM-ECA600 or JEOL JNM-EX400 instrument (JEOL, Tokyo, Japan) using TMS as a reference. Optical rotation was recorded on a JASCO P-2000 polarimeter (Jasco Co., Tokyo, Japan). UV spectrum was obtained using SpectraMax M_2_^e^ spectrophotometer (Molecular Devices, Sunnyvale, CA, USA). IR data were acquired using a Spectrum Jas.co FT/IR-4600 spectrometers (Jasco Corp., Tokyo, Japan). HRESIMS data were obtained using a Waters SYNAPT G2-Si HDMS spectrometer (Waters, Milford, MA, USA).

### 2.2. Plant Material

*Celastrus orbiculatus* (60 kg) was purchased from the Kyung-dong market in Seoul, Korea. One of the authors (M.C. Rho) performed botanical identification, and a voucher specimen (KRIB-KR2016-052) was deposited at the laboratory of the Immunoregulatory Materials Research Center, Jeonbuk Branch of the KRIBB.

### 2.3. Isolation of Compounds **1** and **3**

Pulverized stem of *Celastrus orbiculatus* (60 kg) was extracted at room temperature with 95% EtOH (200 L × 2), and the filtrate was concentrated *in vacuo* to afford the EtOH extract (1.5 kg). The EtOH extract (1.0 kg) was suspended in H_2_O (2.0 L) and subsequently partitioned with *n*-hexane (COH, 225.3 g), EtOAc (COE, 164.9 g), and BuOH (114.4 g) fractions. The EtOAc-soluble extract (130 g) was chromatographed on a silica gel (silica gel, Fuji Silysia Chemical-Chromatorex, 130–200 mesh) column using a step gradient solvent system composed of CHCl_3_ and MeOH (1 : 0 ⟶ 0 : 1, *v*/*v*) to give 17 fractions (COE1–COE17).

COE3 (2.6 g) was subjected to MPLC C_18_ column chromatography (130 g, H_2_O : MeOH = 95 : 5⟶0 : 1, *v*/*v*) to generate 26 subfractions (COE3A–COE3Z). COE3Q (24 mg) was purified by semipreparative HPLC (Phenomenex Luna C_18_, 250 × 21.2 mm, 5 *μ*m, 65% MeCN, 6 mL/min) to obtain compound **1** (12.7 mg, *t*_*R*_ = 33.5 min).

COE5 (4.1 g) was chromatographed on a MPLC silica gel column (120 g, *n*-hexane : EtOAc, 1 : 0 → 0 : 1, *v*/*v*) to yield 15 sub-fractions (COE5A–COE5O), and COE5K (40 mg) was purified by semi-preparative HPLC (YMC, J'sphere ODS H80, 250 ×20 mm, 4 *μ*m, 20% MeOH, 6 mL/min) to give compound **3** (3.4 mg, t*_R_* =54.2 min). Compounds **2** and **4**–**17** were obtained from the hexane-soluble fraction using repeated column chromatography along with EtOAc-soluble fraction (Fig. S1).

Guaiacylglycerol-*α*, *γ*-*O-*nimbidiol diether (**3**) is a white amorphous powder with [*α*]_*D*_^25^ –7 (*c* 0.1, CH_3_OH); UV (CH_3_OH) *λ*_max_ (log *ε*); 221 (4.26), 281 (2.90); IR (ATR) *ν*_max_ 3245, 2963, 2936, 2870, 1652, 1615, 1577, 1511, 1422, 1322, 1251, 1148, 1036, 947, 825 cm^−1^; HRESIMS *m*/*z* 451.2116 [M–H]^–^ (calcd. for C_27_H_31_O_6_^−^, 451.2126). For ^1^H and ^13^C NMR spectroscopic data, see Table 1 (Figs. S2–S16).

### 2.4. Cell Culture

RAW264.7 (ATCC TIB-71) cells was cultured in Dulbecco's modified Eagle medium (DMEM) and RPMI 1640 medium supplemented with 10% fetal bovine serum, 2 mM glutamine, 100 U/mL penicillin, and 100 mg/mL streptomycin sulfate. Cells were maintained at 37°C in humidified air with 5% CO_2_.

### 2.5. Measurement of NO Contents and Cell Cytotoxicity

NO assay was carried out for measurements of NO release using a previously reported method [34]. Briefly, RAW264.7 cells were plated at 1 × 10^5^ cell density in 96-well microplate and cultured for 24 h. Compounds (**1**–**17**) were pretreated with increasing dose concentrations (0.5, 1, 5, 10, 25, 50, and 100 *μ*M) and then stimulated with LPS (1 *μ*g/mL, Sigma–Aldrich, St. Louis, MO, USA) for 18 h. The mixture of cell supernatant (100 *μ*L) and Griess reagent (1% sulfanilamide +0.1% *N*-(1-naphthyl)ethylenediamine (Sigma–Aldrich, St. Louis, MO, USA)) in 5% phosphoric acid was recorded at 550 nm using a microplate reader (Varioskan LUX, Thermo Fisher Scientific Inc., Waltham, MA, USA). The percentage inhibition and logarithmic concentrations were presented as a graph using GraphPad Prism 5 (Fig. S16). IC_50_ values were calculated by nonlinear regression analysis as described previously [35]. RAW264.7 cell cytotoxicity was evaluated using 3-(4,5-dimethylthiazol-2-yl)-2,5-diphenyltetrazolium bromide (MTT) assay [34].

### 2.6. Immunoblot Analysis

The whole cell lysate were extracted using a Cell Lysis Buffer (Cell Signaling Technology, Beverly, MA, USA). Immunoblot analysis was performed using a previously described method [34]. After transfer to nitrocellulose (NC) membrane, the blocking membrane with 5% skimmed milk powder was incubated overnight at 4°C with primary antibody, including anti-phospho-JNK (1 : 1000), anti-JNK (1 : 1000), anti-phospho-p38 (1 : 1000), anti-p38 (1 : 1000), anti-phospho-ERK (1 : 1000), anti-ERK (1 : 1000), anti-phospho-p65 (1 : 1000), anti-p65 (1 : 1000), anti-phospho-I*κ*B*α* (1 : 1000), anti- I*κ*B*α* (1 : 1000), anti-COX-2 (1 : 1000), anti-iNOS (1 : 1000), and anti-*β*-actin antibodies (Cell Signaling, Beverly, MA, USA). The membranes were then incubated with a horseradish peroxide-conjugated anti-rabbit secondary antibody (1 : 5000) at room temperature. The band densities were calculated with Quantity One software (Bio-Rad Laboratories, Hercules, CA, USA).

### 2.7. Real-Time PCR Using TaqMan Probe

Total RNA was extracted from RAW264.7 cells using the TaKaRa MiniBEST Universal RNA Extraction Kit following the manufacturer's instructions (Takara Bio Inc., Japan). The complementary DNA (cDNA) was synthesized from 1 *μ*g of the total RNA using a PrimeScript 1st strand cDNA synthesis kit (Takara Bio Inc. Japan). Quantitative real-time PCR (qPCR) of IL-1b*β* (Mm00434228_m1), IL-6 (Mm00446190_m1), and TNF (Mm00443258_m1) was performed with a TaqMan Gene Expression Assay Kit (Thermo Fisher Scientific, San Jose, CA, USA). To normalize the gene expression, an 18S rRNA endogenous control (Applied Biosystems, Foster City, CA, USA) was used. The qPCR was employed to verify the mRNA expression using a StepOnePlus Real-Time PCR System. To quantify mRNA expression, TaqMan mRNA assay was performed according to the manufacturer's protocol (Applied Biosystems). PCR amplification was analyzed using the comparative ^*ΔΔ*CT^ method.

### 2.8. Statistical Analysis

Half-maximal inhibitory concentration (IC_50_) values expressed as 95% confidence intervals were calculated by nonlinear regression analysis using GraphPad Prism 5 software (GraphPad software, San Diego, CA, USA). Each experiment, including immunoblot and real-time PCR, was performed independently three times, and these data represent the mean ± SEM. The statistical significance of each value was measured by the unpaired Student *t*-test. ^∗^*p* < 0.05, ^∗∗^*p* < 0.01, and ^∗∗∗^*p* < 0.001 were considered significant.

## 3. Results and Discussion

Although *C*. *orbiculatus* is regarded as a medicinal plant including several terpenoids in East Asia and is treated with clinical prescription for health management [11, 36, 37], the biological activity and its composition against the inflammatory action of *C*. *orbiculatus* have hardly been found. In our search for novel anti-inflammatory agents from *C. orbiculatus*, the *n*-hexane and ethyl acetate-soluble fractions of *C*. *orbiculatus* were isolated to yield six diterpenoids (**1**–**6**), nine triterpenoids (**7**–**15**), and two steroids (**16** and **17**) using various column chromatography. Their chemical structures were elucidated as (+)-7-deoxynimbidiol (**1**) [38], nimbidiol (**2**) [39], celaphanol A (**4**) [39], (+)-ferruginol (**5**) [40], dehydroabietic acid (**6**) [41], lupenone (**7**) [42], lupeol (**8**) [42], betulin (**9**) [43], 2*β*,3*β*-dihydroxylup-20(29)-ene (**10**) [44], 3*β*-caffeoyloxylup-20(29)-en-6*α*-ol (**11**) [45], lup-20(29)-en-28-ol-3*β*-yl caffeate (**12**) [43], dammarenediol II 3-caffeate (**13**) [46], *β*-amyrin (**14**) [47], *α*-amyrin (**15**) [47], sitostenon (**16**) [48], and ergone (**17**) [49], compared to previous reported spectroscopic data, NMR, MS, and optical rotation values. Among these, 13 compounds (**3**, **5**–**13**, and **15**–**17**) containing compound 3 determined as novel podocarpane trinorditerpenoid based on HRESIMS and NMR data were first reported from *C*. *orbiculatus* (Figs. S2–S16). The scheme for the isolation of compounds from *Celastrus orbiculatus* was exhibited (Fig. S1).

**Figure 1:**
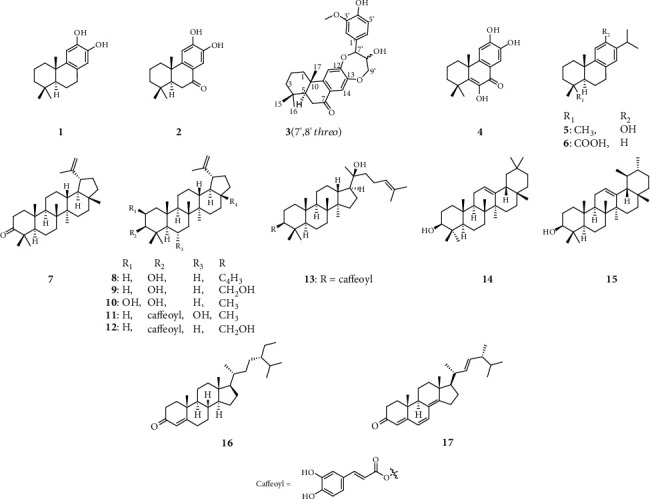
Chemical structure of compounds **1**–**17**.

**Figure 2:**
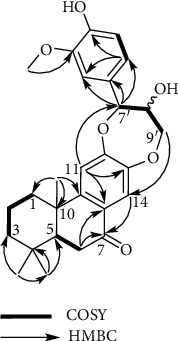
Key COSY and HBMC correlations for compound **3**.

Compound **3** was obtained as white amorphous powder, and its molecular weight of C_27_H_32_O_6_ was determined by HRESIMS deprotonated molecular ion [M–H]^–^ at *m*/*z* 451.2116 (calcd. 451.2126) (Fig. S2). The IR spectrum showed a hydroxy, carbonyl group, and aromatic ring absorption bands (3245, 1652, 1615, 1577, 1511, and 1422 cm^–1^) (Fig. S3). The ^1^H NMR spectrum displayed three methyl protons (*δ*_H_ 0.96/0.95 (s, H_3_-15), 1.03 (s, H_3_-16), and 1.25/1.24 (s, H_3_-17)), two aromatic protons (*δ*_H_ 7.54/7.52 (s, H-14), 6.94/6.93 (s, H-11)), 1,3,4-trisubstituted aromatic ring protons (*δ*_H_ 7.00 (d, *J* = 1.8 Hz, H-2′), 6.84 (d, *J* = 8.4 Hz, H-5′), 6.90 (dd, *J* = 8.4, 1.8 Hz, H-6′)), two oxymethine protons (*δ*_H_ 4.99/4.97 (d, *J* = 8.4 Hz, H-7′), 4.06, (m, H-8′)), one oxymethylene proton (*δ*_H_ 3.71 (dq, *J* = 12.6, 1.2 Hz, H-9′a), 3.47 (dq, *J* = 12.6, 1.8 Hz, H-9′b)), and one methoxy proton (*δ*_H_ 3.88/3.87 (s, OCH_3_-3′)) (Fig. S4). The ^13^C and DEPT NMR spectroscopic data were indicated as the resonance for 27 carbons, including 12 aromatic ring carbons (*δ*_C_ 126.2/126.1 (C-8), 153.0/152.9 (C-9), 113.4/113.3 (C-11), 151.2/151.1 (C-12), 143.6/143.5 (C-13), 116.4 (C-14), 129.0/128.9 (C-1′), 112.2/112.1 (C-2′), 149.4 (C-3′), 148.7 (C-4′), 116.5 (C-5′), and 121.9 (C-6′)), three methyl carbons (*δ*_C_ 33.2 (C-15), 21.9 (C-16), and 23.9/23.8 (C-17)), four methylene carbons (*δ*_C_ 39.2 (C-1), 20.1 (C-2), 42.7 (C-3), 37.1/37.0 (C-6)), one oxymethylene carbon (*δ*_C_ 62.1 (C-9′)), one methine carbon (*δ*_C_ 51.4/51.3 (C-5)), two oxymethine carbons (*δ*_C_ 78.7/78.6 (C-7′), 80.0/79.9 (C-8′)), two quaternary carbons (*δ*_C_ 34.3 (C-4), 39.4/39.3 (C-10)), methoxy carbon (*δ*_C_ 56.6 (OCH_3_-3′)), and carbonyl carbon (*δ*_C_ 200.5 (C-7)) (Fig. S4 and S5). Its 1D NMR data closely resembled that of nimbidiol (**2**), which is previously isolated from *Celastrus* genus [39], except for the additional guaiacylglycerol group based on key COSY (H-7′/H-8′/H_2_-9′) and HMBC (H-7′/C-1′, -2′, -3′ and OCH_3_-3/C-3′) correlations (Figs. S8 and S10). The positions of *α* and *γ* in the guaiacylglycerol group were determined to be located at OH-12 and OH-13 of nimbidiol moiety, respectively, which involved a diether moiety, on the basis of the long range correlations (HMBC) between H-11 and C-7′ (*α*) and between H_2_-9 (*γ*) and C-14 (Figure 1 and Fig. S10). The relative configuration of **3** was elucidated to be the same as that of nimbidiol by NOESY correlation between H-5 and H_3_-15 and between H_3_-16/H_3_-17. Furthermore, the large coupling constant for *J*_7′/8′_ (8.4 Hz) in the guaiacylglycerol group and no observation of NOE correlation between H-7′ and H-8′ indicated relative *threo* configuration (Fig. S11). Therefore, a pair of 1D NMR spectra of the same pattern showed that ′ is a 1 : 1 mixture of *threo* isomers between C-7′ and -8′. The structure of **3** was elucidated as shown in Figure 2, named guaiacylglycerol-*α*, *γ*-*O-*nimbidiol diether.

In maintenance of homeostasis from various organs systems, NO has been recognized as one of the important biological mediator involved in the various pathophysiological and physiological mechanisms, such as neurotransmitters, host defense against pathogenic microorganism, and regulation of immune systems [50]. However, the overproduction of NO in intracellular levels is associated to inflammatory diseases and carcinogenesis, and measurement of NO content has been employed by various literatures on the anti-inflammatory properties of phytochemicals derived from natural products [51]. To investigate whether NO production stimulated by LPS was inhibited by phytochemicals isolated from *C*. *orbiculatus*, compounds **1**–**17** were tested by NO assay in the RAW264.7 cells. As shown in Table 2, **1**–**4**, **11**, and **12** showed potent inhibitory activity against LPS-treated NO secretion based on 50% inhibitory effect at 50 *μ*M concentration compared to only LPS-treated control group (IC_50_: 4.9–40.0 *μ*M) (Fig. S17), and all isolates did not affect cytotoxicity at IC_50_ concentration, respectively (Fig. S18). Among isolates showing NO inhibitory effect, **1** and **3**, which are podocarpane-type trinorditerpenoid class, were selected to evaluate further anti-inflammatory activity at 5 or 10 *μ*M concentrations, respectively, which are approximately IC_50_values without cytotoxicity effect by compounds.

iNOS is a major downstream mediator of inflammation in several cell types including macrophage cells [52]. During the course of an inflammatory response, large amount of NO formed by the action of iNOS surpass the physiological amounts of NO [53], and consequentially, iNOS overproduction reflects the degree of inflammation [54, 55]. COX-2 is an inducible enzyme that has a role in the development of epithelial cell dysplasia, carcinoma, wound edge of tissue, and inflammatory diseases such as arthritis, allergic asthma, and atopic dermatitis [56–58]. The expression of COX-2 is a key mediator of inflammatory pathway, which is representatively the NF-*κ*B signaling pathway [59, 60].

**Figure 3:**
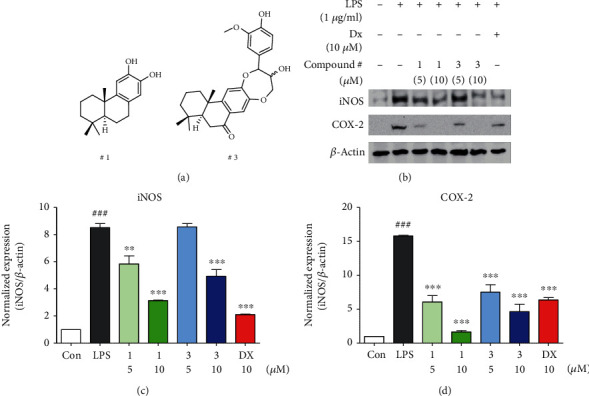
Compounds **1** and **3** showed anti-inflammatory effects through inhibiting iNOS and COX-2. (a) Chemical structure of compounds **1** and **3**. (b) Compounds **1** and **3** decreased iNOS and COX-2 protein expression levels in LPS-induced RAW264.7 cells. (c, d) Relative ratio of iNOS and COX-2 versus *β*-actin was measured using densitometry, and dexamethasone was used as positive control. These graphs represented that compounds **1** and **3** dose dependently inhibited iNOS and COX-2 levels in immunoblot analysis. Cells were pretreated with each compound for 2 h and stimulated with LPS (1 *μ*g/mL) for 16 h. Immunoblot analysis performed a triplicate test, and results are expressed as means ± SEM. An unpaired Student *t*-test was used for statistical analysis. ^###^*p* < 0.001, ^∗∗^*p* < 0.01, and ^∗∗∗^*p* < 0.001 versus LPS.

In order to examine the biological evidence of effectively reduced NO production after treatment with **1** and **3**, we performed the immunoblot analysis to investigate whether **1** and **3** suppressed the upregulation of iNOS and COX-2 protein expression after LPS-activated inflammation condition. As shown in Figure 3, **1** and **3** dose dependently inhibited iNOS and COX-2 protein expression on LPS-induced inflammation in RAW264.7 cells. In addition, a comparison of nitric oxide production between compound **1**, **3**, and celastrol was exhibited (Fig. S19).

Each protein expression level was represented as relative ratio values of iNOS/*β*-actin and COX-2/*β*-actin (Figures 3(c) and 3(d)). The fold-change values in iNOS and COX-2 expression in the presence of **1** and **3** was as follows: control (1 ± 0), LPS (8.51 ± 0.51/15.82 ± 0.15), **1** (5 *μ*M: 5.84 ± 1.02/6.08 ± 1.61 and 10 *μ*M: 3.13 ± 0.05/1.65 ± 0.34), **3** (5 *μ*M: 8.55 ± 0.44/7.53 ± 1.88 and 10 *μ*M: 4.91 ± 0.86/4.66 ± 1.84), and dexamethasone (10 *μ*M: 2.1 ± 0.06/6.38 ± 0.59). These results suggested that **1** and **3** prevented NO production via inhibition iNOS and COX-2 expression under LPS-induced inflammation condition in macrophages.

Dexamethasone or nonsteroidal anti-inflammatory drugs (NSAIDs) [61] are well known for blocking the MAPKs and NF-*κ*B signaling cascades and results in potent anti-inflammatory activity through the reduction of proinflammatory mediators such as iNOS and COX-2. MAPK (JNK, ERK, and p38) and NF-*κ*B are crucial intracellular signaling pathways leading to the inflammatory response. These biological response are mediated by their transcription factors, such as activator protein- (AP-) 1, cAMP response element-binding protein (CREB), and NF-*κ*B, which are phosphorylated and activated in the cytoplasmic or nuclear, resulting in an inflammatory action via the expression of target genes including proinflammatory cytokines IL-1*β*, IL-6, and TNF-*α* as well as iNOS and COX-2 proteins [62–64].

**Figure 4:**
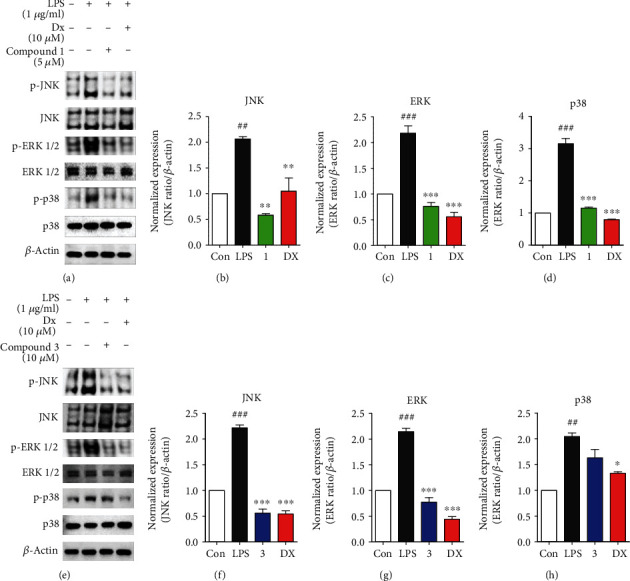
Compounds **1** and **3** suppressed MAPK signaling pathway. (a, e) Immunoblot analysis showed that phosphorylated protein levels of MAPK signaling cascades, JNK, ERK1/2, and p38 are inhibited by compounds **1** (a) and **3** (e) in RAW264.7 macrophages. (b–d, f–h) Total-JNK, ERK1/2, and p38 MAPK proteins were used as loading controls. (b, f) Cells were preincubated for 2 h with each compound **1** and **3** at concentrations of 5 and 10 *μ*M, respectively, and stimulated with LPS (1 *μ*g/mL) for 1 h. Dexamethasone served as the positive control. Immunoblot analysis performed triplicate experiments, and data represented means ± SEM. Significant difference was considered at the levels of ^##^*p* < 0.01, ^###^*p* < 0.001, ^∗^*p* < 0.05, ^∗∗^*p* < 0.01, and ^∗∗∗^*p* < 0.001 versus LPS.

**Figure 5:**
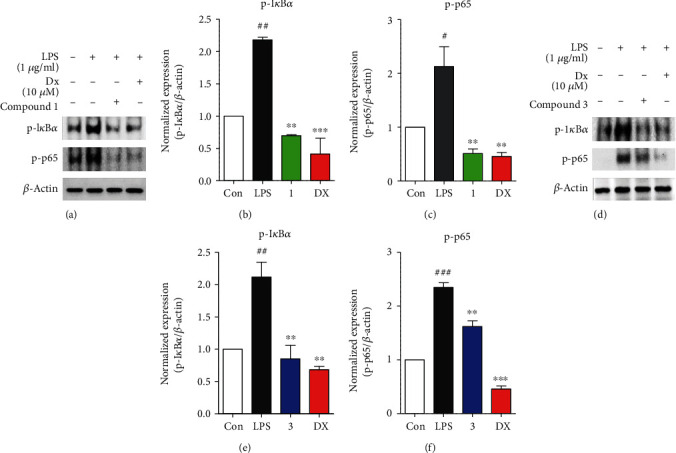
Compounds **1** and **3** attenuated the NF-*κ*B signaling pathway. (a, d) Immunoblot analysis displayed that activation of the NF-*κ*B signaling pathway was suppressed by compounds **1** (a) and **3** (d) in RAW264.7 cells. (b, c, e, f) The graph was expressed as the values of the relative ratio I*κ*B*α* or p65 to *β*-actin protein expression level using densitometry. Cells were pretreated for 2 h with compounds **1** and **3** at concentrations of 5 and 10 *μ*M, respectively, and stimulated with LPS (1 *μ*g/mL) for 1 h. Dexamethasone was used as the positive control, and immunoblots analysis performed triplicate experiments. Values are means ± SEM, and an unpaired Student *t*-test was used for statistical analysis. ^#^*p* < 0.05, ^##^*p* < 0.01, ^###^*p* < 0.001, ^∗^*p* < 0.05, ^∗∗^*p* < 0.01, and ^∗∗∗^*p* < 0.001 represented significant differences from the LPS-treated group.

To further investigate anti-inflammatory effects associated with inhibition of NO production, iNOS, and COX-2, major inflammatory signaling cascades, MAPKs (JNK, ERK, and p38), and NF-*κ*B, were evaluated with treatment of **1** or **3** in LPS-induced murine macrophages. As shown in Figures 4(a)–4(d), **1** remarkably inhibited phosphorylation of JNK (p-JNK), ERK (p-ERK), and p38 (p-p38) MAPK signaling molecules on LPS-activated inflammatory condition in RAW264.7 cells. Each protein expression level was presented as relative ratio values of p-JNK/JNK, p-ERK/ERK, and p-p38/p38. The fold-change values in p-JNK, p-ERK, and p-p38 expression in the presence of **1** were as follows: control (1 ± 0), LPS (2.06 ± 0.07/2.18 ± 0.24/3.15 ± 0.27), **1** (5 *μ*M: 0.58 ± 0.05/0.76 ± 0.12/1.14 ± 0.05), and dexamethasone (10 *μ*M: 1.04 ± 0.44/0.55 ± 0.15/0.79 ± 0.02). As shown in Figures 4(e)–4(h), **3** markedly suppressed p-JNK and p-ERK, but not p-p38. The fold-change values in p-JNK, ERK, and p-p38 expression in the presence of **3** were as follows: control (1 ± 0), LPS (2.21 ± 0.09/2.14 ± 0.11/2.04 ± 0.11), **3** (10 *μ*M: 0.56 ± 0.13/0.77 ± 0.15/1.63 ± 0.28), and dexamethasone (10 *μ*M: 0.54 ± 0.05/0.44 ± 0.08/1.32 ± 0.05). Subsequently, immunoblot analysis was used to examine whether **1** and **3** affect the activation of NF-*κ*B transcription factor through a decrease of phosphorylation of I*κ*B*α* (p-I*κ*B*α*) and p65 (p-p65). **1** and **3** significantly inhibited p-I*κ*B*α* and p-p65, similar to the positive control, dexamethasone (Figure 5). Each protein expression level was expressed as relative ratio values of p-I*κ*B*α*/*β*-actin and p-p65/*β*-actin as described in Figures 5(b), 5(c), 5(e), and 5(f). The fold-change values in p-I*κ*B*α* and p-p65 expression in the presence of **1** were as follows: control (1 ± 0), LPS (2.17 ± 0.07/2.13 ± 0.63), **1** (5 *μ*M: 0.69 ± 0.02/0.51 ± 0.14), and dexamethasone (10 *μ*M: 0.41 ± 0.42/0.45 ± 0.12) (Figures 5(b) and 5(c)). The fold-change values in p-I*κ*B*α* and p-p65 expression in the presence of **3** were as follows: control (1 ± 0), LPS (2.21 ± 0.09/2.34 ± 0.15), **3** (10 *μ*M: 0.56 ± 0.13/1.62 ± 0.18), and dexamethasone (10 *μ*M: 0.54 ± 0.05/0.45 ± 0.09) (Figure 5(e) and 5(f)). These results suggested that the anti-inflammatory activity of **1** and **3** is responsible for suppressing the MAPK and NF-*κ*B signaling pathways.

**Figure 6:**
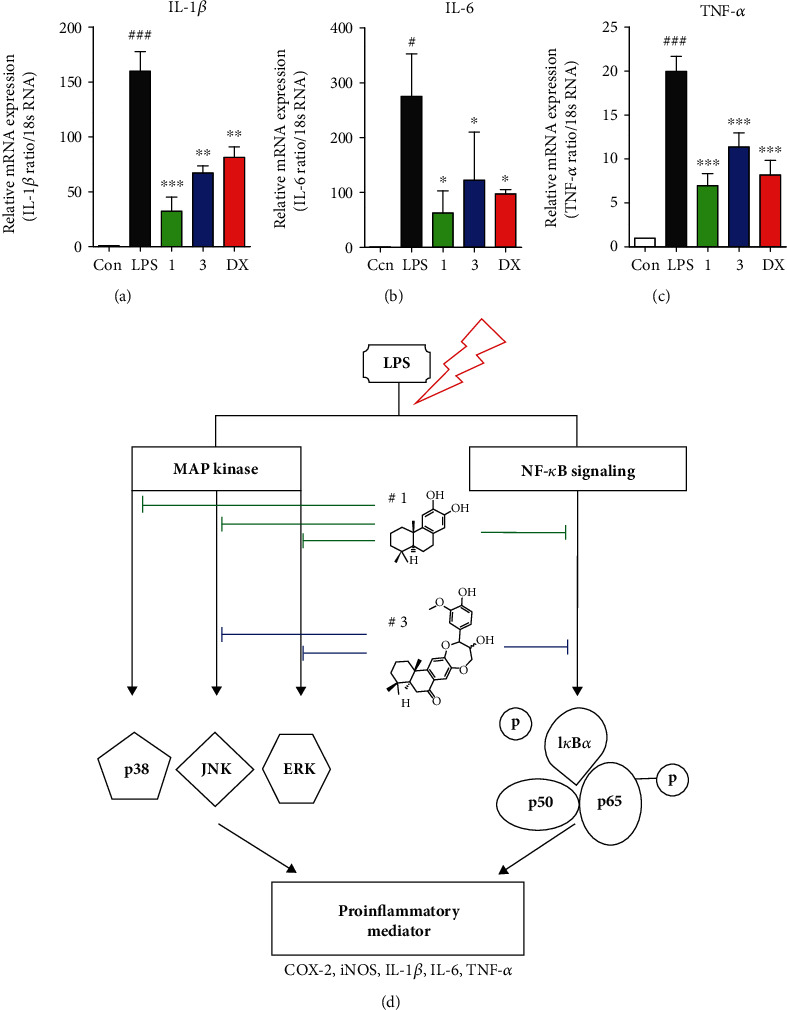
Compounds **1** and **3** downregulated proinflammatory mediators. (a–c) The mRNA expression levels of IL-1*β*, IL-6, and TNF-*α* were measured using quantitative real-time PCR experiment, and these proinflammatory cytokines were significantly diminished by compounds **1** and **3**. Cells were preincubated for 2 h with compounds **1** and **3** at concentration of 5 and 10 *μ*M, respectively, and activated by LPS (1 *μ*g/mL) for 2 h. Results represent as mean ± SEM, and dexamethasone was used as a positive control. ^#^*p* < 0.05, ^###^*p* < 0.001, ^∗^*p* < 0.05, ^∗∗^*p* < 0.01, and ^∗∗∗^*p* < 0.001 indicated significant differences from the LPS-treated group. (d) Graphical depiction of the potent anti-inflammatory activity of compounds **1** and **3** in LPS-activated RAW264.7 cells by suppressing the MAPK and NF-*κ*B signaling pathway.

The continuous overexpression of proinflammatory cytokines, IL-1*β*, IL-6, and TNF-*α*, is characterized as chronic inflammatory pathogenesis, which results in cell and tissue degeneration [63, 65], such as rheumatoid arthritis and inflammatory bowel diseases. Thus, following the hypothesis that these proinflammatory cytokines may be inhibited by **1** and **3**, we performed real-time PCR experiments to evaluate the inhibitory effect of IL-1*β*, IL-6, and TNF-*α* levels. In accordance with our hypothesis, **1** and **3** revealed a reduction in LPS-induced IL-1*β*, IL-6, and TNF-*α* gene expression at mRNA transcription levels (Figure 6). All taken together, these results indicated that the anti-inflammation activity of **1** and **3** was attributed to blockade of the MAPK and NF-*κ*B signaling pathways via the suppression of p-ERK, p-JNK, p-p38, p-I*κ*B, and p-p65 (Figure 6(d)).

## 4. Conclusion

In the present study, compounds **1**–**17** separated from *C*. *orbiculatus* using normal or reverse phase column chromatography were identified as six diterpenoids (**1**–**6**), nine triterpenoids (**7**–**15**), and two steroids (**16** and **17**) compared to previous reported spectroscopic data including NMR and MS. Of all isolates, 7-deoxynimbidiol (**1**) and novel podocarpane-type trinorditerpenoid (**3**) significantly exhibited the most significant inhibitory effects on LPS-activated proinflammatory mediator secretion, such as iNOS, COX-2, NO, IL-1*β*, IL-6, and TNF-*α*, and its anti-inflammatory actions were exerted via downregulation of MAPK and NF-*κ*B signaling cascade molecules including p-ERK, p-JNK, p-p38, p-I*κ*B, and p-p65. Therefore, *C*. *orbiculatus* extract and its components **1** and **3** may be useful and safe treatments for inflammatory diseases such as rheumatoid arthritis, asthma, and atopic dermatitis, which can be applied to an alternative medical food in place of the conventional drugs, such as NSAIDs and dexamethasone.

## Figures and Tables

**Table 1:** ^1^H and ^13^C NMR spectroscopic data (*δ* ppm) for compound **3**.

Position	**3**		
	*δ*_C_^a^		*δ*_H_^b^ (*J* in Hz)
1	39.2	CH_2_	2.29, d (12.6)
			1.50, m
2	20.1	CH_2_	1.83^a^, m
			1.67, br d (13.8)
3	42.7	CH_2_	1.55, d (13.2)
			1.32, td (13.2, 2.4)
4	34.3	C	—
5	51.4/51.3	CH	1.84^a^, m
6	37.1/37.0	CH_2_	2.64, m
7	200.5	C	—
8	126.2/126.1	C	—
9	153.0/152.9	C	—
10	39.4/39.3	C	—
11	113.4/113.3	CH	6.94^a^, s/6.93^a^, s
12	151.2/151.1	C	—
13	143.6/143.5	C	—
14	116.4	CH	7.54, s/7.52, s
15	33.2	CH_3_	0.96^a^, s/0.95ª, s
16	21.9	CH_3_	1.03, s
17	23.9/23.8	CH_3_	1.25^a^, s/1.24^a^, s
1'	129.0/128.9	C	—
2'	112.2/112.1	CH	7.00, d (1.8)
3'	149.4	C	—
4'	148.7	C	—
5'	116.5	CH	6.84, d (8.4)
6'	121.9	CH	6.90, dd (8.4, 1.8)
7'	78.7/78.6	CH	4.99, d (8.4)/4.97, d (8.4)
8'	80.0/79.9	CH	4.06, tdd (8.4, 4.2, 2.4)
9'	62.1	CH_2_	3.71, ddd (12.6, 2.4, 1.2)
			3.47, ddd (12.6, 4.2, 1.8)
OCH_3_-3'	56.6	CH_3_	3.88, s/3.87, s

Assignments were done by HSQC, HMBC, and COSY experiments. Spectra were measured in methanol-*d*_4_ at 600 and 150 MHz. ^a^Overlapped signals.

**Table 2:** Inhibitory effects of compounds (**1**–**17**) on LPS-induced NO production.

Compound	IC_50_ (*μ*M)	Compound	IC_50_ (*μ*M)
**1**	4.89 (4.77–5.01)	**10**	>50
**2**	38.72 (17.50–85.66)	**11**	18.07 (10.74–30.42)
**3**	12.60 (10.65–14.89)	**12**	39.99 (30.42–52.58)
**4**	13.13 (9.15–18.84)	**13**	>50
**5**	>50	**14**	>50
**6**	>50	**15**	>50
**7**	>50	**16**	>50
**8**	>50	**17**	>50
**9**	>50	**Dexamethasone** ^a^	0.016 (0.011– 0.023)

The IC_50_ values are showed with 95% confidence intervals (95% CIs). ^a^Cytotoxicity was not observed at the IC_50_ concentration. ^b^Dexamethasone used as the positive control.

## Data Availability

The data used to support the findings of this study are available from the corresponding author upon request.

## Supplementary Materials

Supplementary Materials Supplementary Figure 1: scheme for the isolation of compounds from Celastrus orbiculatus. Supplementary Figure 2: HRESIMS spectrum of **3**. Supplementary Figure 3: IR spectrum of **3**. Supplementary Figure 4: ^1^H NMR (600 MHz, methanol-d4) spectrum of **3**. Supplementary Figure 5: ^13^C NMR (150 MHz, methanol-d4) spectrum of **3**. Supplementary Figure 6: DEPT90 NMR (150 MHz, methanol-d4) spectrum of **3**. Supplementary Figure 7: DEPT-135 NMR (150 MHz, methanol-d4) spectrum of **3**. Supplementary Figure 8: COSY (600 MHz, methanold4) spectrum of **3**. Supplementary Figure 9: HMQC (600 MHz, methanol-d4) spectrum of **3**. Supplementary Figure 10: HMBC (600 MHz, methanol-d4) spectrum of **3**. Supplementary Figure 11: NOESY (600 MHz, methanol-d4) spectrum of **3**. Supplementary Figure 12: ^1^H NMR (600 MHz, DMSO-d6) spectrum of **3**. Supplementary Figure 13: ^13^C NMR (150 MHz, DMSOd6) spectrum of **3**. Supplementary Figure 14: COSY (600 MHz, DMSO-d6) spectrum of **3**. Supplementary Figure 15: HMQC (600 MHz, DMSO-d6) spectrum of **3**. Supplementary Figure 16: HMBC (600 MHz, DMSO-d4) spectrum of **3**. Supplementary Figure 17: inhibition percentage curves for the compounds **1**–**4**, **11**, and **12**. Supplementary Figure 18: cell viability 17 for the compounds **1**–**4**, **11**, and **12**. Supplementary Figure 19: a comparison of Nitric oxide production between compounds **1**, **3**, and celastrol.
