# Supplementary material for: Anti-Inflammatory Activity of Diterpenoids from Celastrus orbiculatus in Lipopolysaccharide-Stimulated RAW264.7 Cells
Source: J Immunol Res. 2020 Jul 30;2020:7207354. doi: 10.1155/2020/7207354 (PMC7414338; doi:10.1155/2020/7207354)
Supplement: Supplementary Materials — Supplementary Figure 1: scheme for the isolation of compounds from Celastrus orbiculatus. Supplementary Figure 2: HRESIMS spectrum of 3. Supplementary Figure 3: IR spectrum of 3. Supplementary Figure 4: 1H NMR (600 MHz, methanol-d4) spectrum of 3. Supplementary Figure 5: 13C NMR (150 MHz, methanol-d4) spectrum of 3. Supplementary Figure 6: DEPT90 NMR (150 MHz, methanol-d4) spectrum of 3. Supplementary Figure 7: DEPT-135 NMR (150 MHz, methanol-d4) spectrum of 3. Supplementary Figure 8: COSY (600 MHz, methanold4) spectrum of 3. Supplementary Figure 9: HMQC (600 MHz, methanol-d4) spectrum of 3. Supplementary Figure 10: HMBC (600 MHz, methanol-d4) spectrum of 3. Supplementary Figure 11: NOESY (600 MHz, methanol-d4) spectrum of 3. Supplementary Figure 12: 1H NMR (600 MHz, DMSO-d6) spectrum of 3. Supplementary Figure 13: 13C NMR (150 MHz, DMSOd6) spectrum of 3. Supplementary Figure 14: COSY (600 MHz, DMSO-d6) spectrum of 3. Supplementary Figure 15: HMQC (600 MHz, DMSO-d6) spectrum of 3. Supplementary Figure 16: HMBC (600 MHz, DMSO-d4) spectrum of 3. Supplementary Figure 17: inhibition percentage curves for the compounds 1–4, 11, and 12. Supplementary Figure 18: cell viability 17 for the compounds 1–4, 11, and 12. Supplementary Figure 19: a comparison of Nitric oxide production between compounds 1, 3, and celastrol. [file 7207354.f1.pdf]

Supplementary Data

Anti-inflammatory activity of diterpenoids from *Celastrus orbiculatus* in lipopolysaccharide-stimulated RAW264.7 cells

Hyun-Jae Jang[a,1], Kang-Hoon Kim[a,1], Eun-Jae Park [a,b], Jeong A Kang[a,b], Bong-Sik Yun[b], Seung-Jae Lee [a], Chan Sun Park [a], Soyoung Lee [a], Seung Woong Lee [a,*], Mun-Chual Rho [a,*]

[a]*Immunoregulatory Materials Research Center, Korea Research Institute of Bioscience and Biotechnology, 181 Ipsin-gil, Jeon geup-si, Jeonbuk 56212, Korea*

[b]*Division of Biotechnology and Advanced Institute of Environment and Bioscience, College of Environmental and Bioresource Sciences, Chonbuk National University, Iksan-si, Republic of Korea*

[1] These authors equally contributed to this study.

Corresponding Authors

*(S. Lee) Tel: 82-63-570-5264. Fax: 82-63-570-5239. E-mail: lswdoc@kribb.re.kr

Content		Page
Further purification of compounds **1–17**		S1, S2
**Figure S1.**	Scheme for the isolation of compounds from *Celastrus orbiculatus*	S3
**Figure S2.**	HRESIMS spectrum of **3**	S4
**Figure S3**	IR spectrum of **3**	S5
**Figure S4**	^{1}H NMR (600 MHz, methanol-d_4) spectrum of **3**	S6
**Figure S5**	^{13}C NMR (150 MHz, methanol-d_4) spectrum of **3**	S7
**Figure S6**	DEPT-90 NMR (150 MHz, methanol-d_4) spectrum of **3**	S8
**Figure S7**	DEPT-135 NMR (150 MHz, methanol-d_4) spectrum of **3**	S9
**Figure S8**	COSY (600 MHz, methanol-d_4) spectrum of **3**	S10
**Figure S9.**	HMQC (600 MHz, methanol-d_4) spectrum of **3**	S11
**Figure S10.**	HMBC (600 MHz, methanol-d_4) spectrum of **3**	S12
**Figure S11.**	NOESY (600 MHz, methanol-d_4) spectrum of **3**	S13
**Figure S12.**	^{1}H NMR (600 MHz, DMSO-d_6) spectrum of **3**	S14
**Figure S13.**	^{13}C NMR (150 MHz, DMSO-d_6) spectrum of **3**	S15
**Figure S14.**	COSY (600 MHz, DMSO-d_6) spectrum of **3**	S16
**Figure S15.**	HMQC (600 MHz, DMSO-d_6) spectrum of **3**	S17
**Figure S16.**	HMBC (600 MHz, DMSO-d_6) spectrum of **3**	S18
**Figure S17.**	Inhibition percentage curves for the compounds **1–4**, **11**, and **12**	S19
**Figure S18.**	Cell viability for the compounds **1–4**, **11**, and **12**	S20
**Figure S19.**	A comparison of Nitric oxide production between compound **1**, **3**, and celastrol	S21

*Further purification of compounds **2**, **4–17***

The *n*-hexane-soluble extract (200 g) was separated by silica gel column chromatography (silica gel, Fuji Silysia Chemical-Chromatorex, 130–200 mesh) using a gradient solvent system of *n*-hexane:EtOAc (1:0 → 0:1, v/v) to afford eight sub-fractions (COH1–COH8). COH3 (9.1 g) was subjected to silica gel column chromatography using the gradient solvent of *n*-hexane:acetone (1:0 → 0:1, v/v) to give 20 sub-fraction (COH3A–COH3T). COH3F (3.4 g) was applied to MPLC equipped with a C_{18} column (RediSep C_{18} column, 130 g) and eluted with H_2O:MeOH (4:6 → 0:1, v/v) to produce 13sub-fractions (COH3F1–COH3F13). COH3F6 (20.4 mg) was separated by semi-preparative HPLC (Phenomenex Luna C_{18}, 250 × 21.2 mm, 5 μm, 90% MeCN, 6 mL/min) to obtain **5** (15.8 mg, t_R = 54.9 min). COH3F10 (200 mg) was recrystallized in MeOH to obtain **7** (131.5 mg). COH5 (21.2 g) was subjected to MPLC (RediSep silica column 120 g) using the gradient solvent of *n*-hexane:acetone (1:0 → 0:1, v/v) to give 20 sub-fractions (COH5A–COH5P). COH5C (3.1 g) was fractionation by MPLC (H_2O:MeOH = 4:6 → 0:1, v/v) equipped with a C_{18} column (130 g) to give 16 sub-fractions (COH5C1–COH5C16). COH5C5 (7.5 mg) was further purified by semi-preparative HPLC (Phenomenex Luna C_{18}, 250 × 21.2 mm, 5 μm, 65% MeCN, 6 mL/min) to give **6** (2.1 mg, t_R = 60.2 min). **17** (10.1 mg, t_R = 75.2 min) and **8** (16.5 mg, t_R = 70.8 min) were isolated from COH5C10 (63 mg) and COH5C11 (400 mg) using semi-preparative HPLC (Phenomenex Kinetex C_{18}, 250 × 21.2 mm, 5 μm, 90% and 92% MeCN, 6 mL/min), respectively. COH5C12 (500 mg) was separated by MPLC (RediSep silica column, 40 g) using a gradient solvent of *n*-nexane:EtOAc (1:0 → 50:1, v/v) to generate 10 sub-fractions (COH5C12A–COH5C12J). Semi-preparative HPLC (Phenomenex Luna C_8, 250 × 21.2 mm, 5 μm, 95% MeCN, 6 mL/min) was used

to separate **14** (5.8 mg, t_R = 53.1 min) and **15** (5.1 mg, t_R = 56.2 min) from COH5C12D and purify **16** (21 mg, t_R = 58.3 min) from COH5C12E. COH6 (6.4 g) was chromatographed on a MPLC silica column (silica column, 120 g, *n*-hexane:acetone = 1:0 → 0:1, v/v) to yield 15 sub-fractions (COH6A–COH6O). COH6D (4.4 g) was applied to MPLC (silica column, 120 g, *n*-hexane:EtOAc = 1:0 → 0:1, v/v) to obtain 18 sub-fractions (COH6D1 – COH6D18), and COH6D11 (502 mg) was isolated using semi-preparative HPLC (Phenomenex Luna C_8, 150 × 21.2 mm, 5 μm, 97% MeCN, 6 mL/min) to yield **10** (21.9 mg, t_R = 38.2 min). COH6F (2.7 g) was subjected to MPLC C_{18} column chromatography (130 g, H_2O:MeOH = 6:4 → 0:1, v/v) to generate nine sub-fractions (COH6F1–COH6F9). COH6F9 (87 mg) was further separated using semi-preparative HPLC (Phenomenex Kinetex C_{18}, 150 × 21.2 mm, 5 μm, 75% MeCN, 6 mL/min) to afford **9** (10.5 mg, t_R = 45.2 min). The EtOAc-soluble extract (130 g) was chromatographed on a silica gel column using a step gradient solvent system composed of $CHCl_3$ and MeOH (1:0 → 0:1, v/v) to give 17 fractions (COE1–COE17). COE3 (2.6 g) was subjected to MPLC C_{18} column chromatography (130 g, H_2O:MeOH = 95:5 → 0:1, v/v) to generate 26 sub-fractions (COE3A–COE3Z). **11** (29 mg, t_R = 48.7 min) and **13** (5.9 mg, t_R = 40.2 min) were further separated from COE3S (70 mg) using semi-preparative HPLC (YMC J'sphere ODS H-80, 150 × 20 mm, 4 μm, 98% MeCN, 6 mL/min). COE7 (4.4 g) was subjected by MPLC (C_{18}, 130 g, H_2O:MeOH = 95:5 → 0:1, v/v) to generate 21 sub-fractions (COE7A–COE7U). **2** (30.2 mg, t_R = 36.7 min) and **4** (78.4 mg, t_R = 50.1 min) were isolated from COE7K (205.8 mg) using semi-preparative HPLC (YMC J'sphere ODS H-80, 150 × 20 mm, 4 μm, 45% MeCN, 6 mL/min), and **12** (27.3 mg, t_R = 50.4 min) was separated from COE7R (190.3 mg) using semi-preparative HPLC (YMC J'sphere ODS H-80, 150 × 20 mm, 4 μm, 75% MeCN, 6 mL/min).

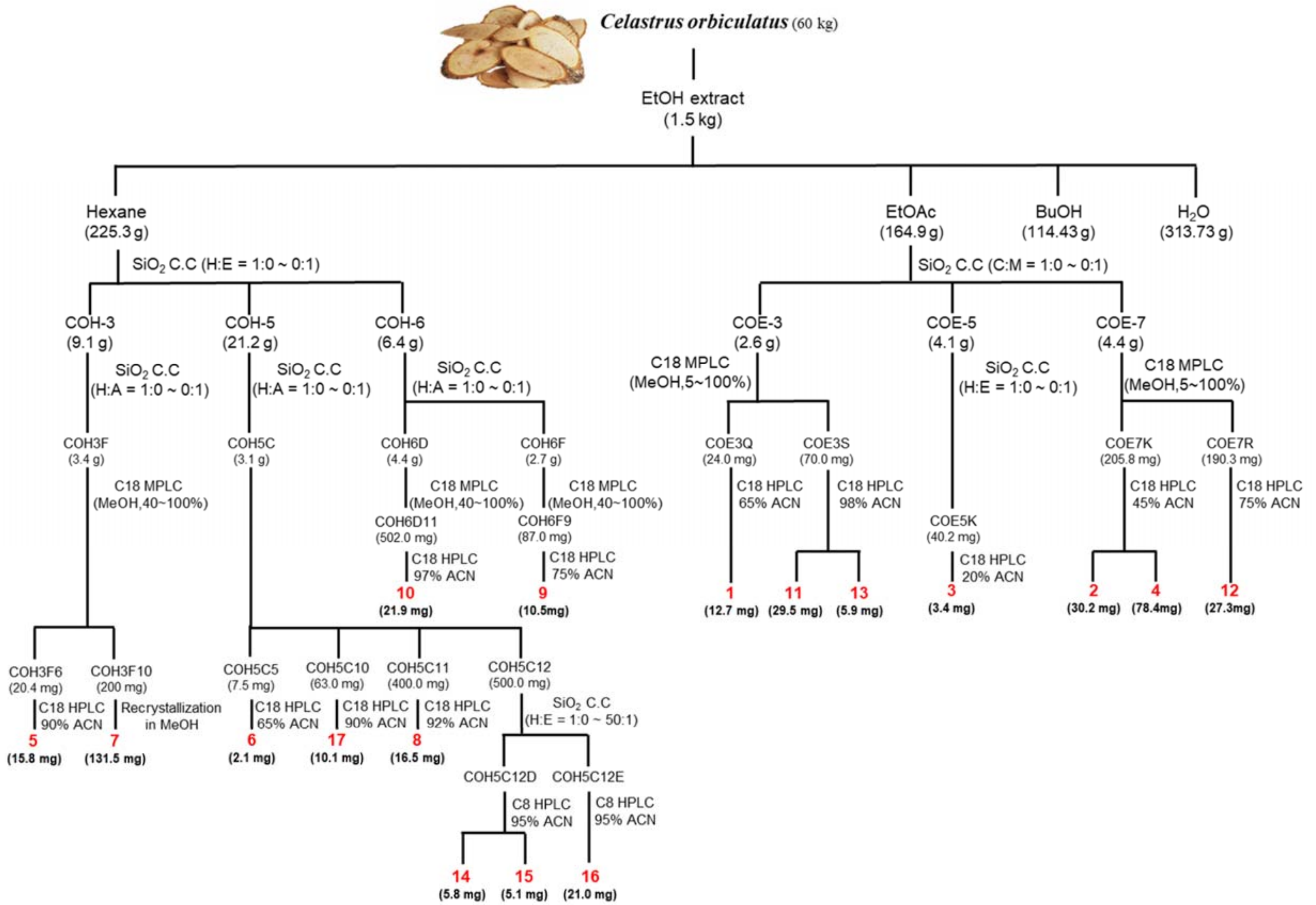

Figure S1. Scheme for the isolation of compounds **1–17** from *Celastrus orbiculatus*

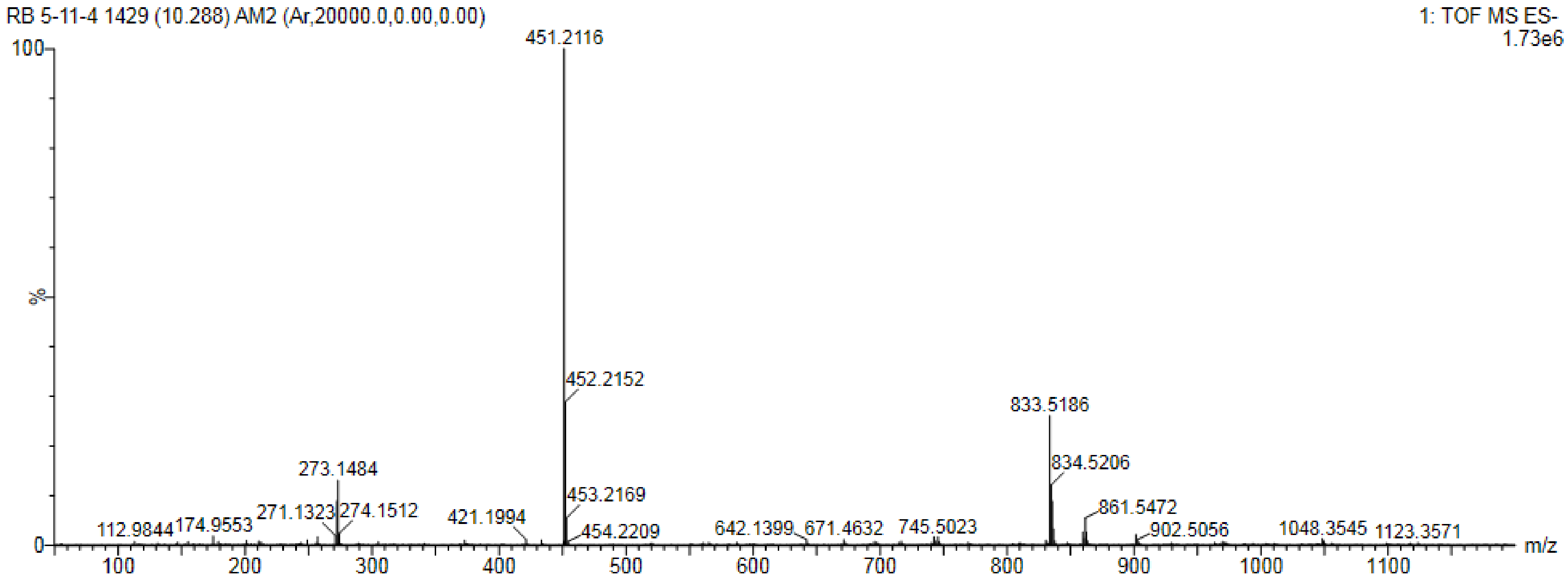

Figure S2. HRESIMS spectrum of **3**

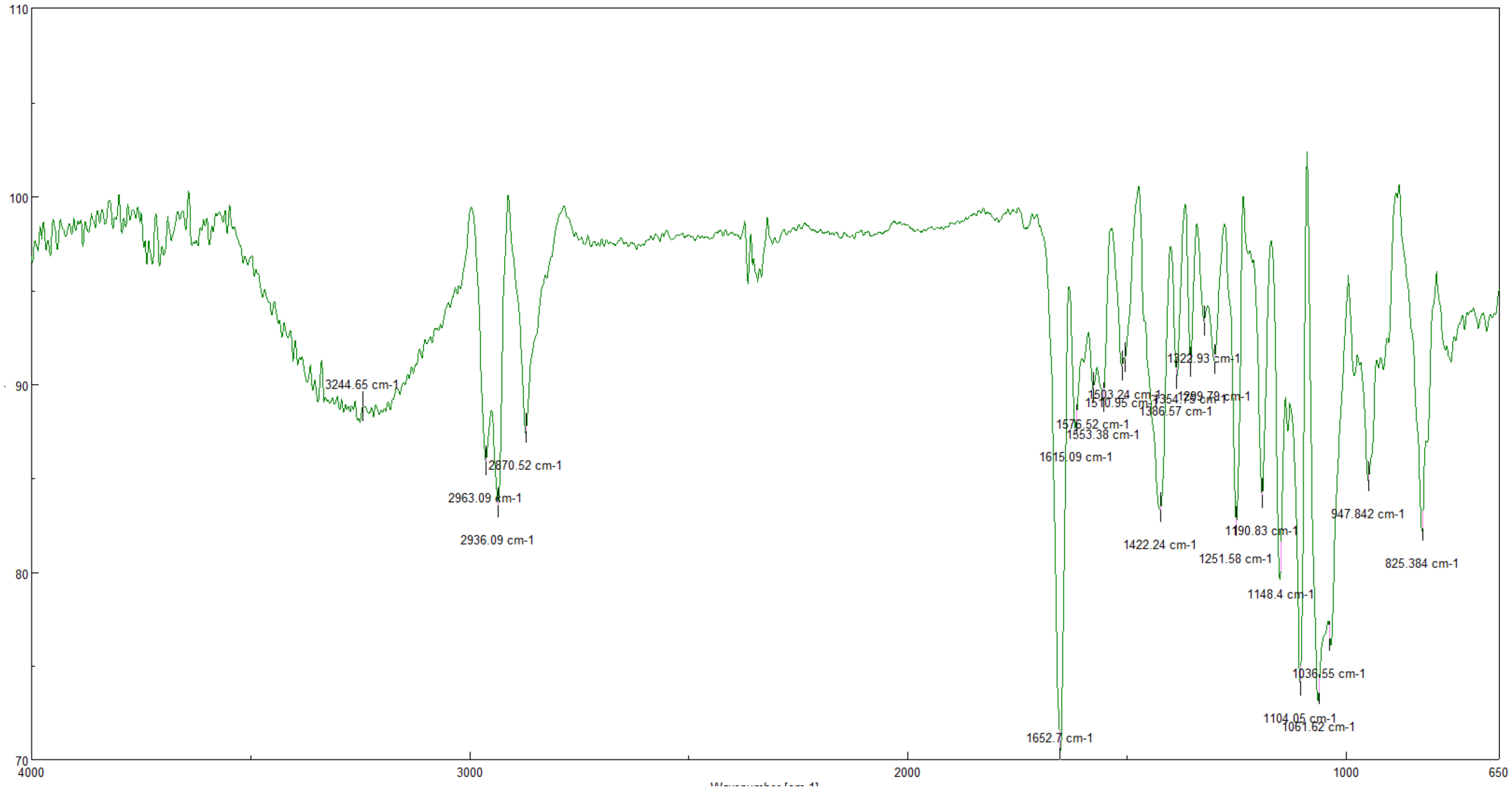

Figure S3. IR spectrum of **3**

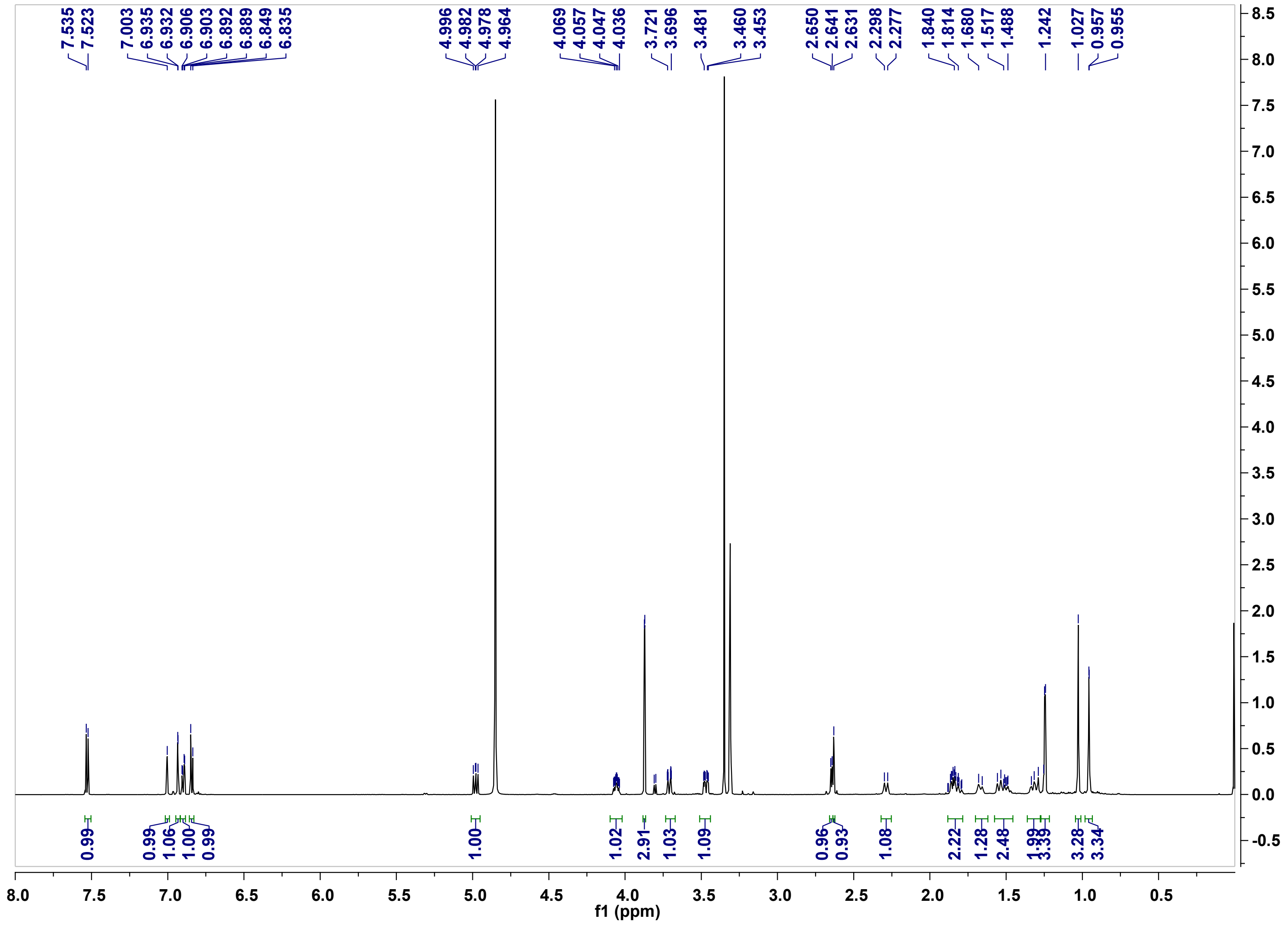

Figure S4. ^{1}H NMR (600 MHz, methanol-d_4) spectrum of **3**

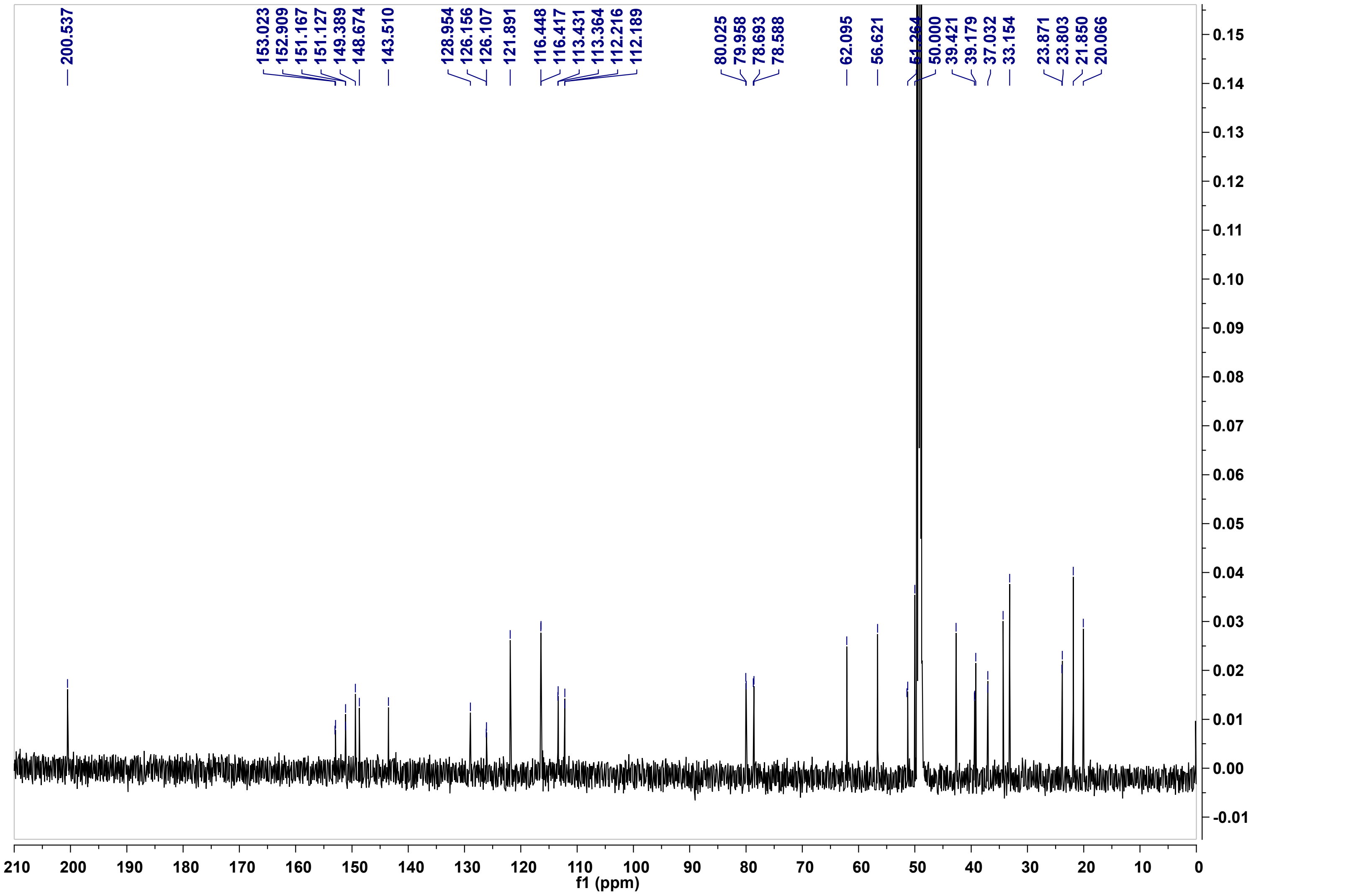

Figure S5. ^{13}C NMR (150 MHz, methanol-d_4) spectrum of **3**

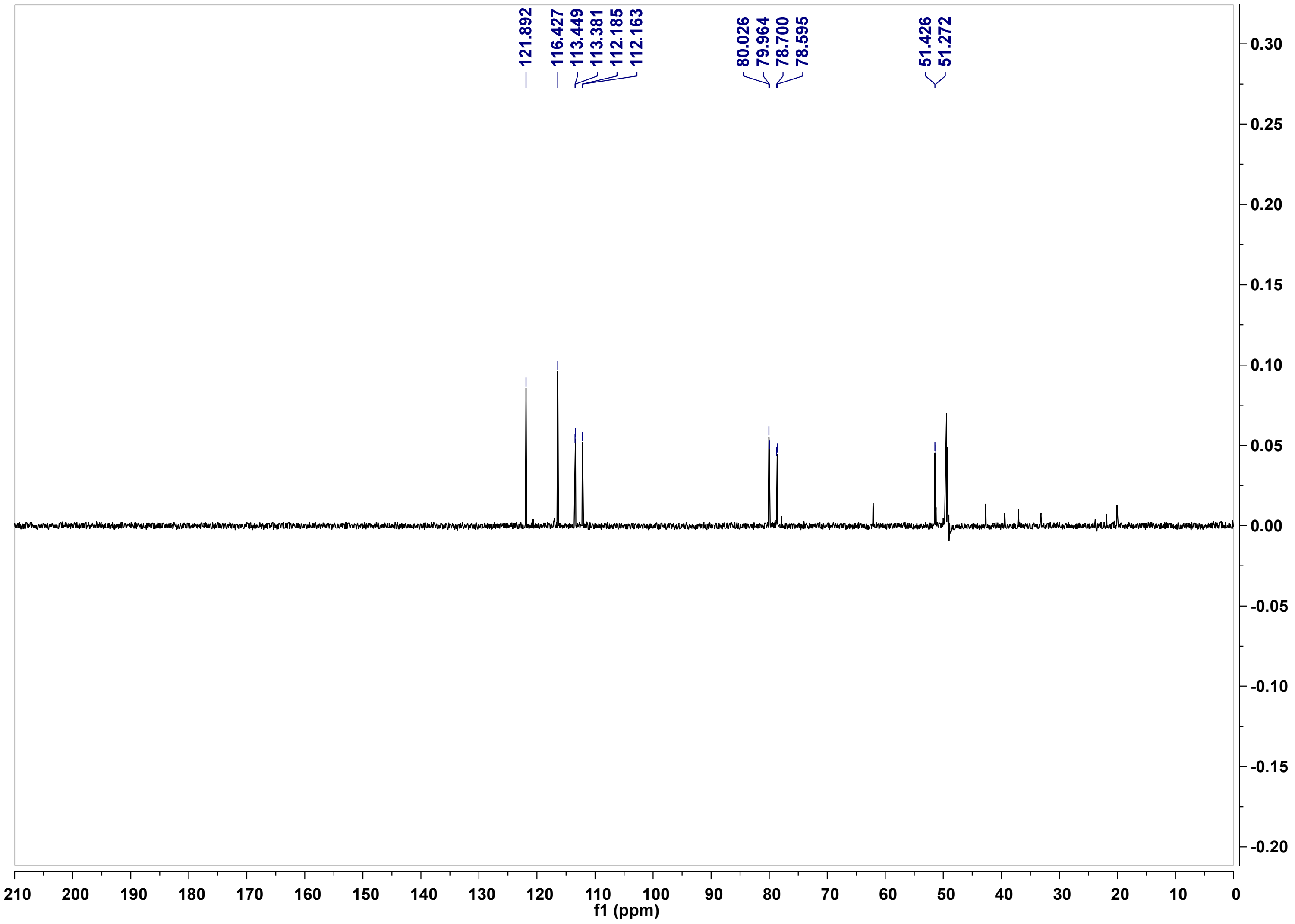

Figure S6. DEPT-90 NMR (150 MHz, methanol-*d*4) spectrum of **3**

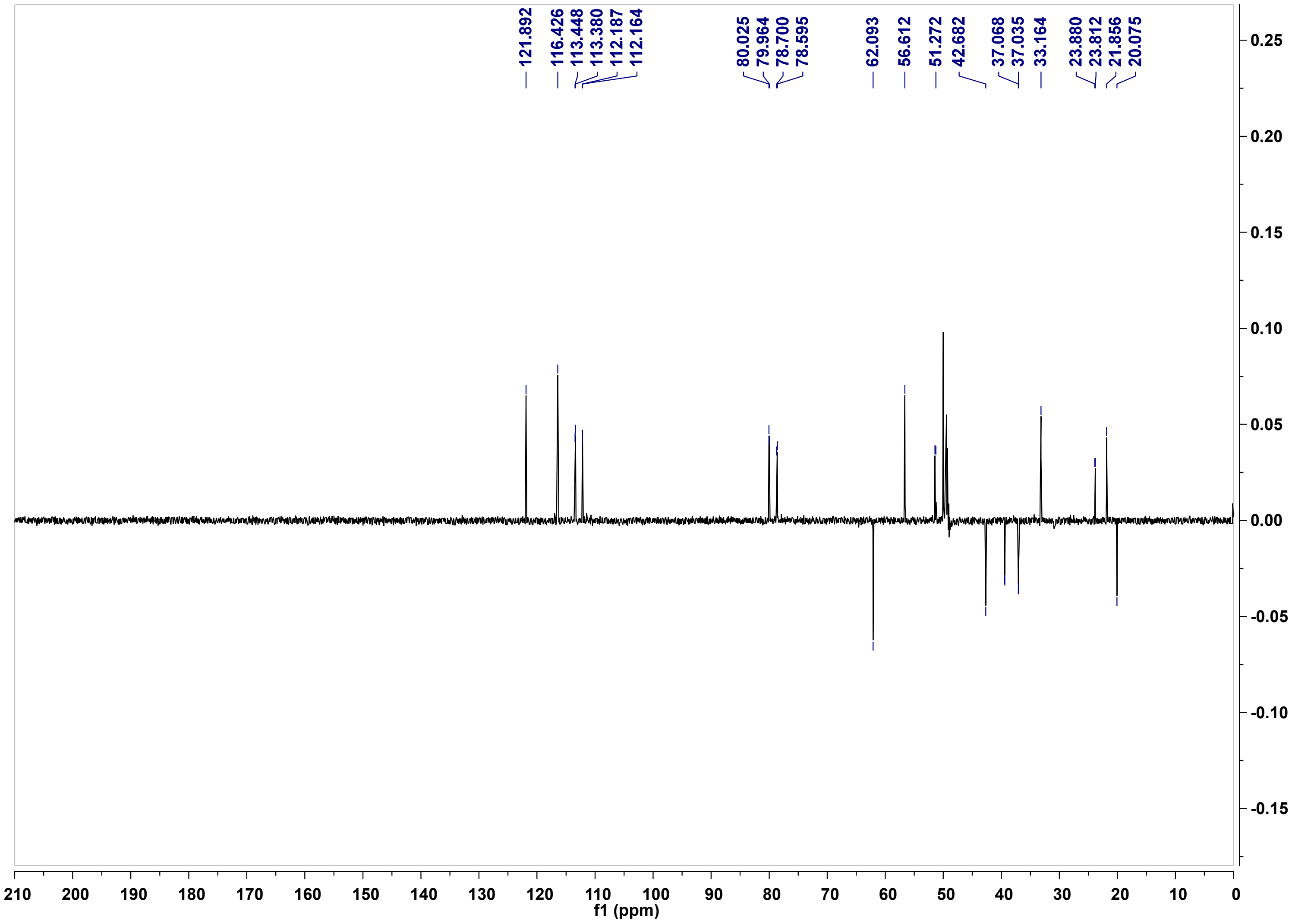

Figure S7. DEPT-135 NMR (150 MHz, methanol-d_4) spectrum of **3**

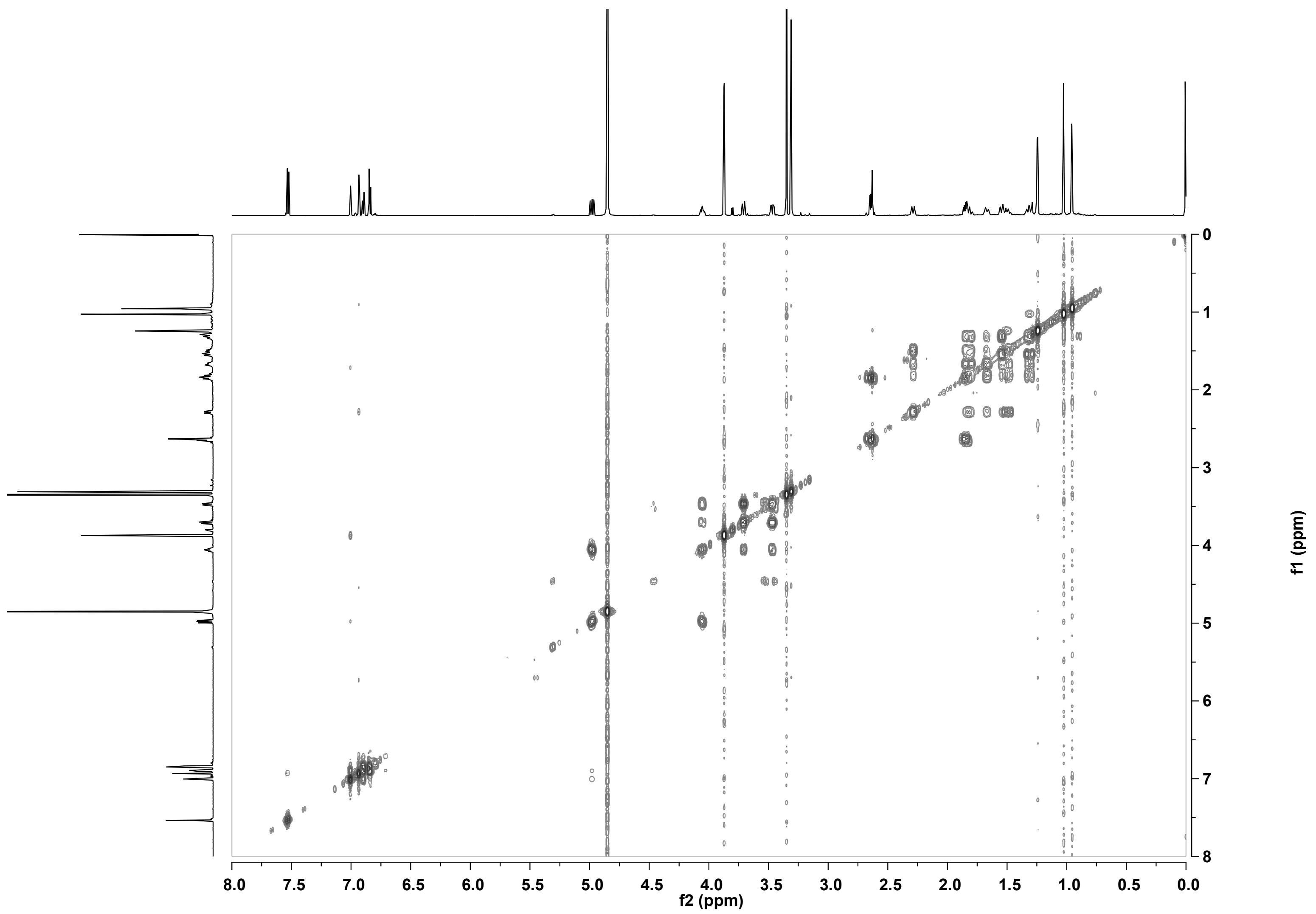

Figure S8. COSY (600 MHz, methanol-d_4) spectrum of **3**

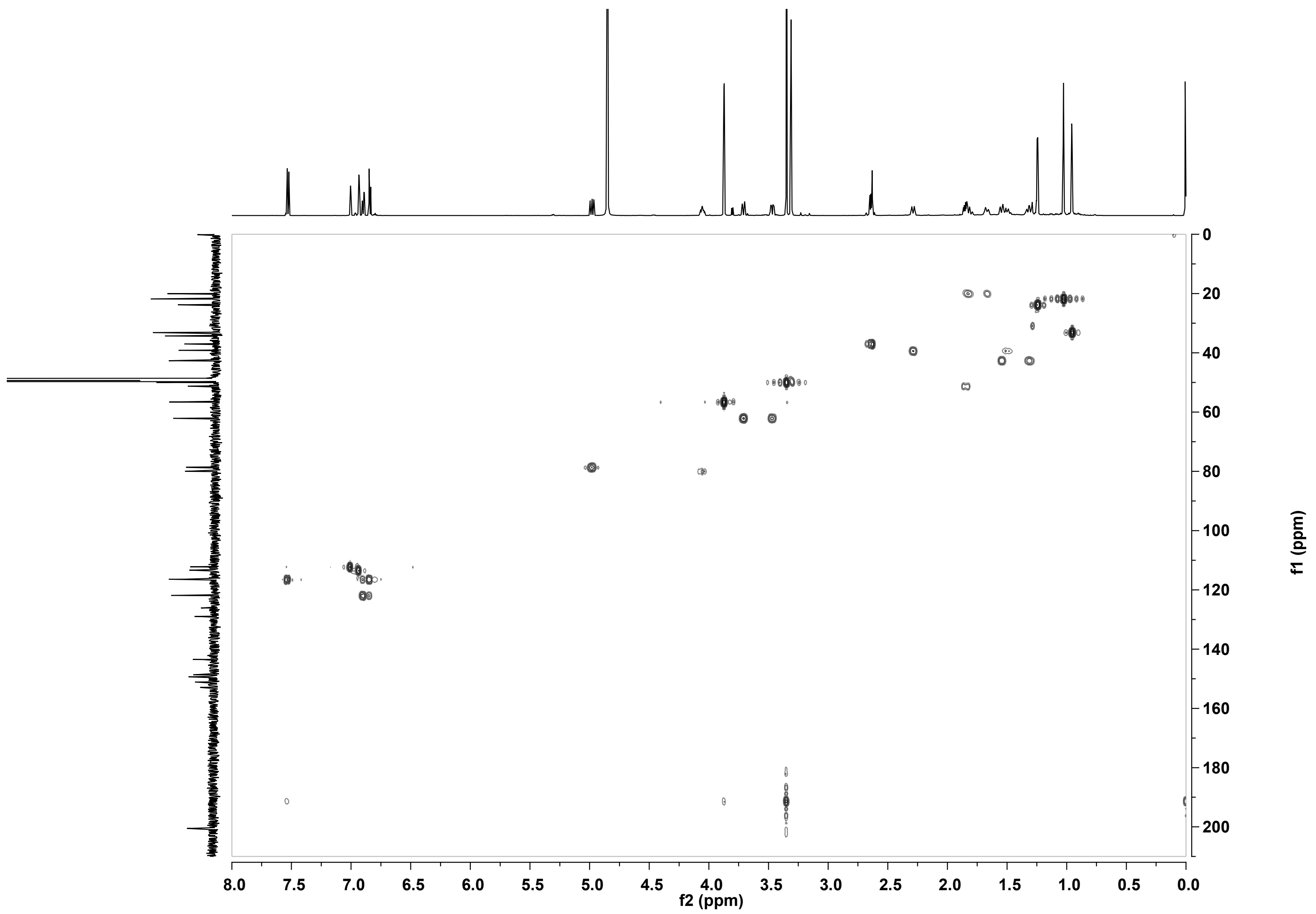

Figure S9. HMQC (600 MHz, methanol-d_4) spectrum of **3**

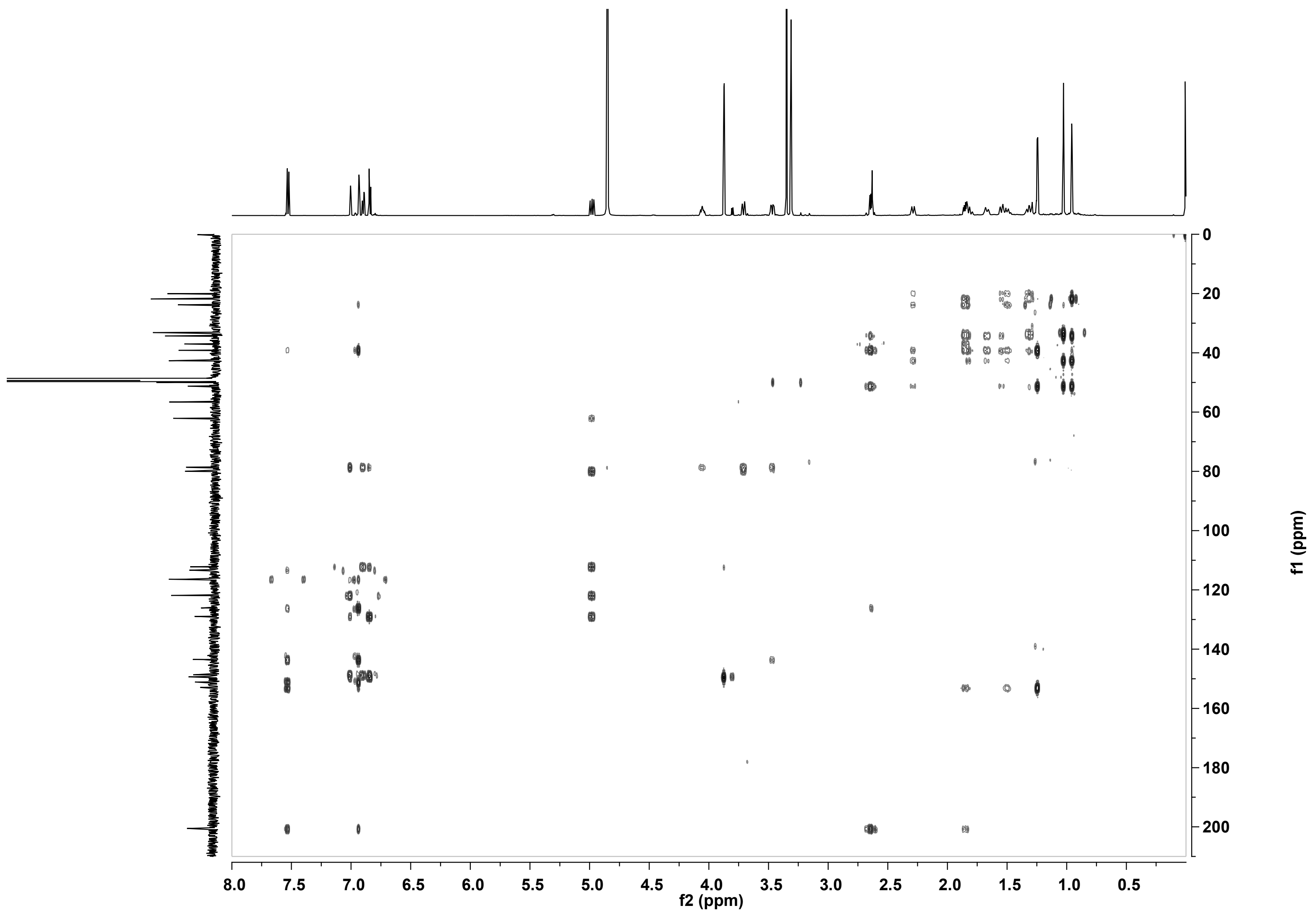

Figure S10. HMBC (600 MHz, methanol-d_4) spectrum of **3**

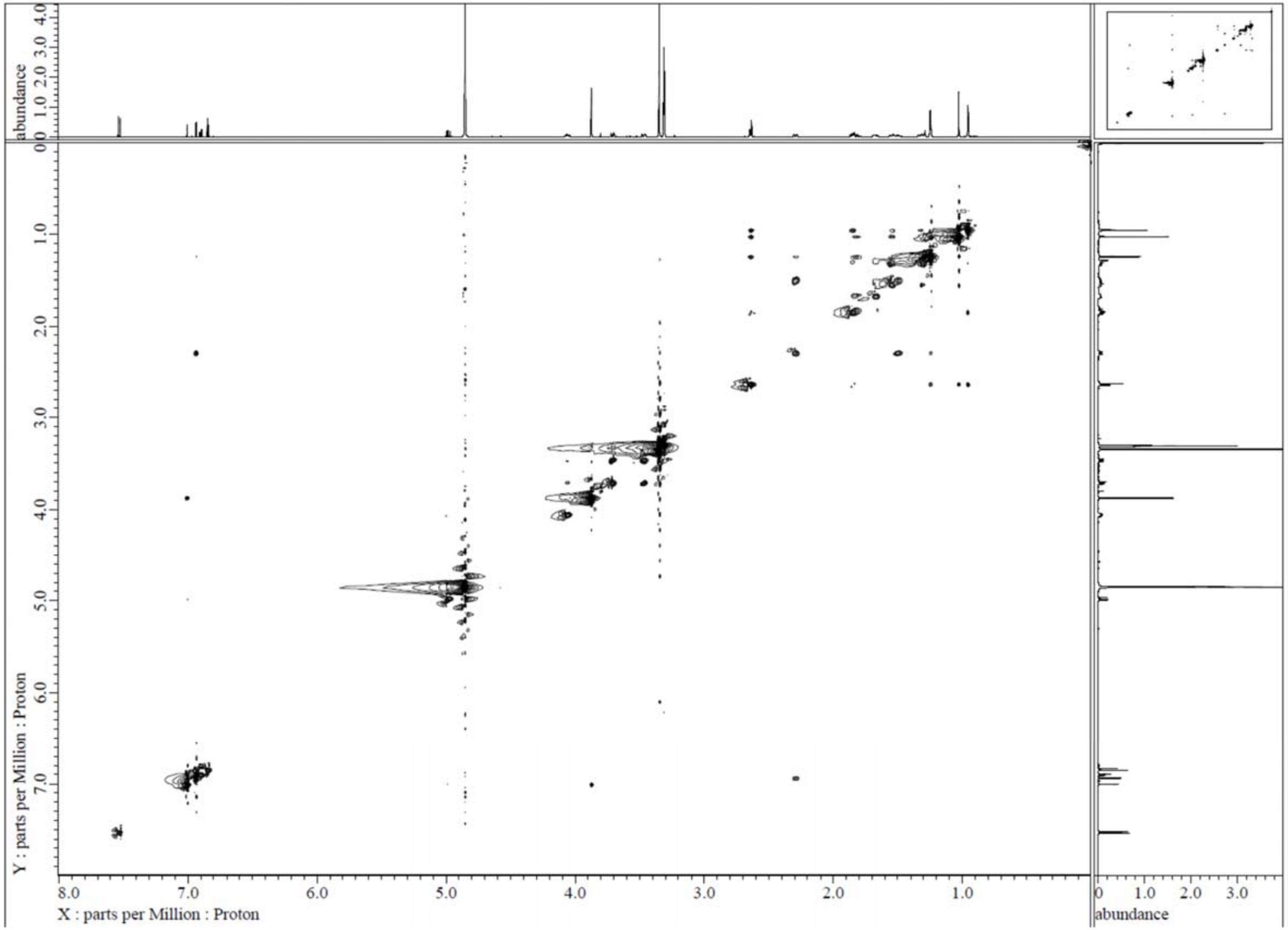

Figure S11. NOESY (600 MHz, methanol-d_4) spectrum of **3**

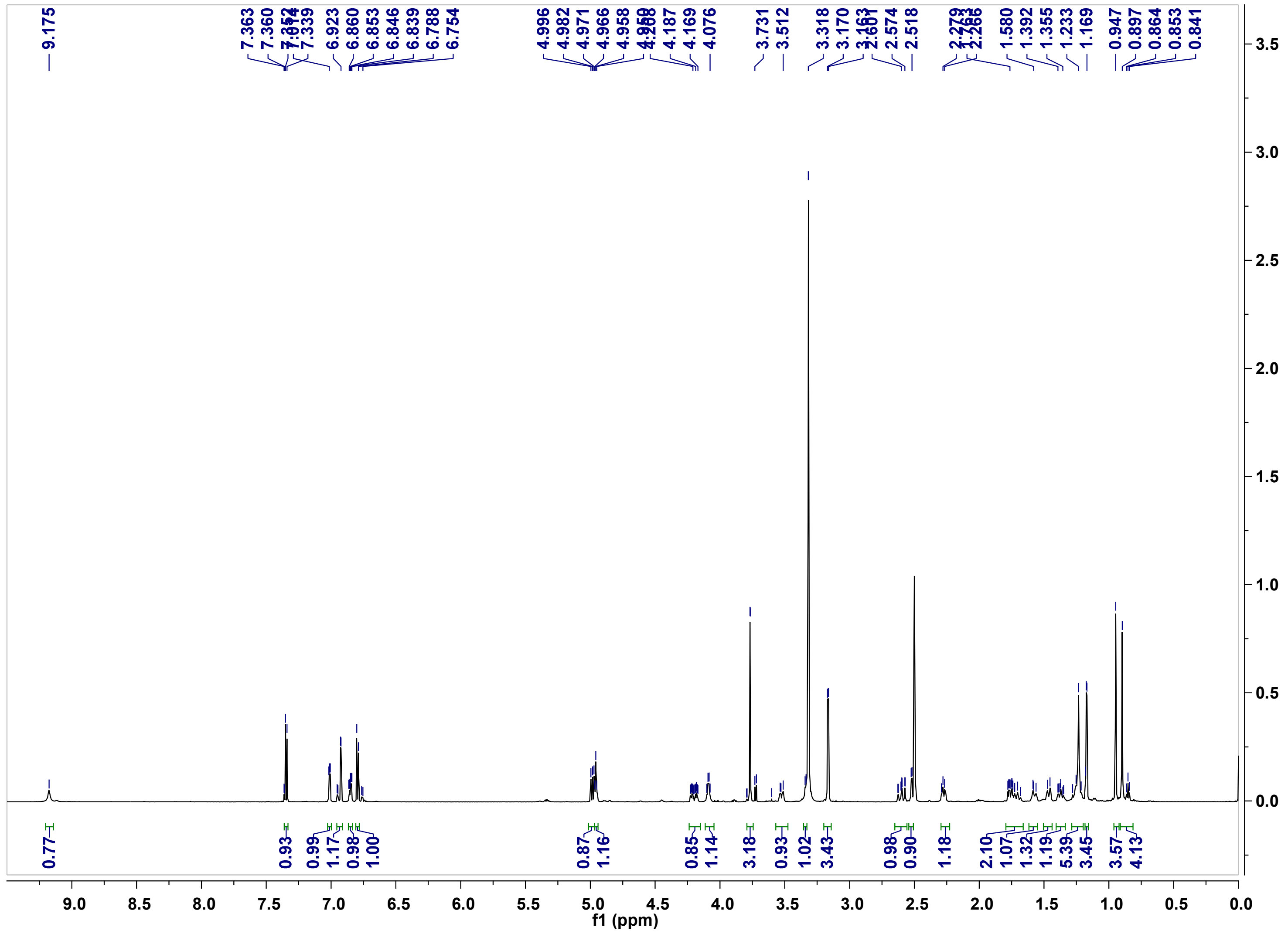

Figure S12. ^{1}H NMR (600 MHz, DMSO-d_6) spectrum of **3**

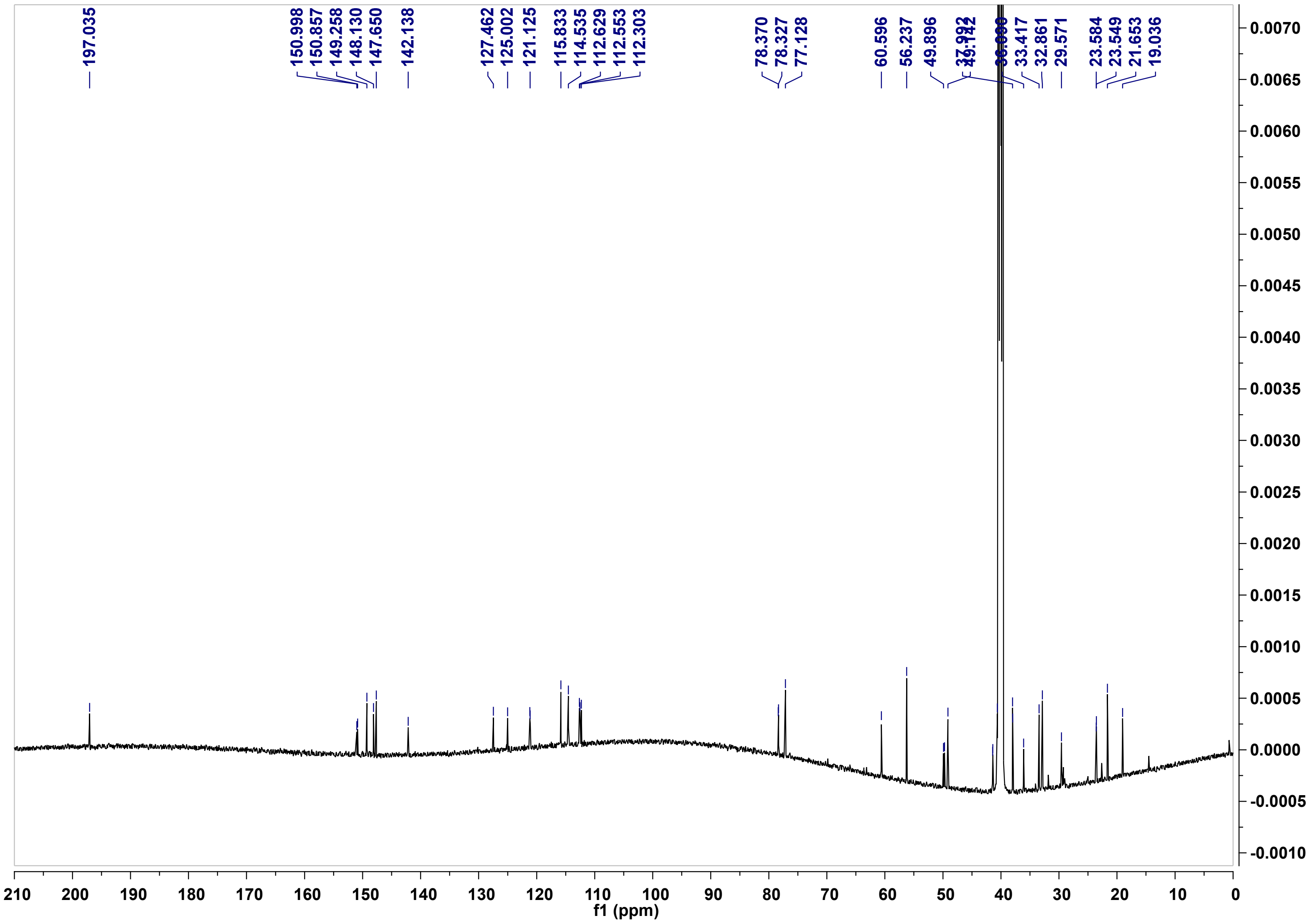

Figure S13. ^{13}C NMR (150 MHz, DMSO-d_6) spectrum of **3**

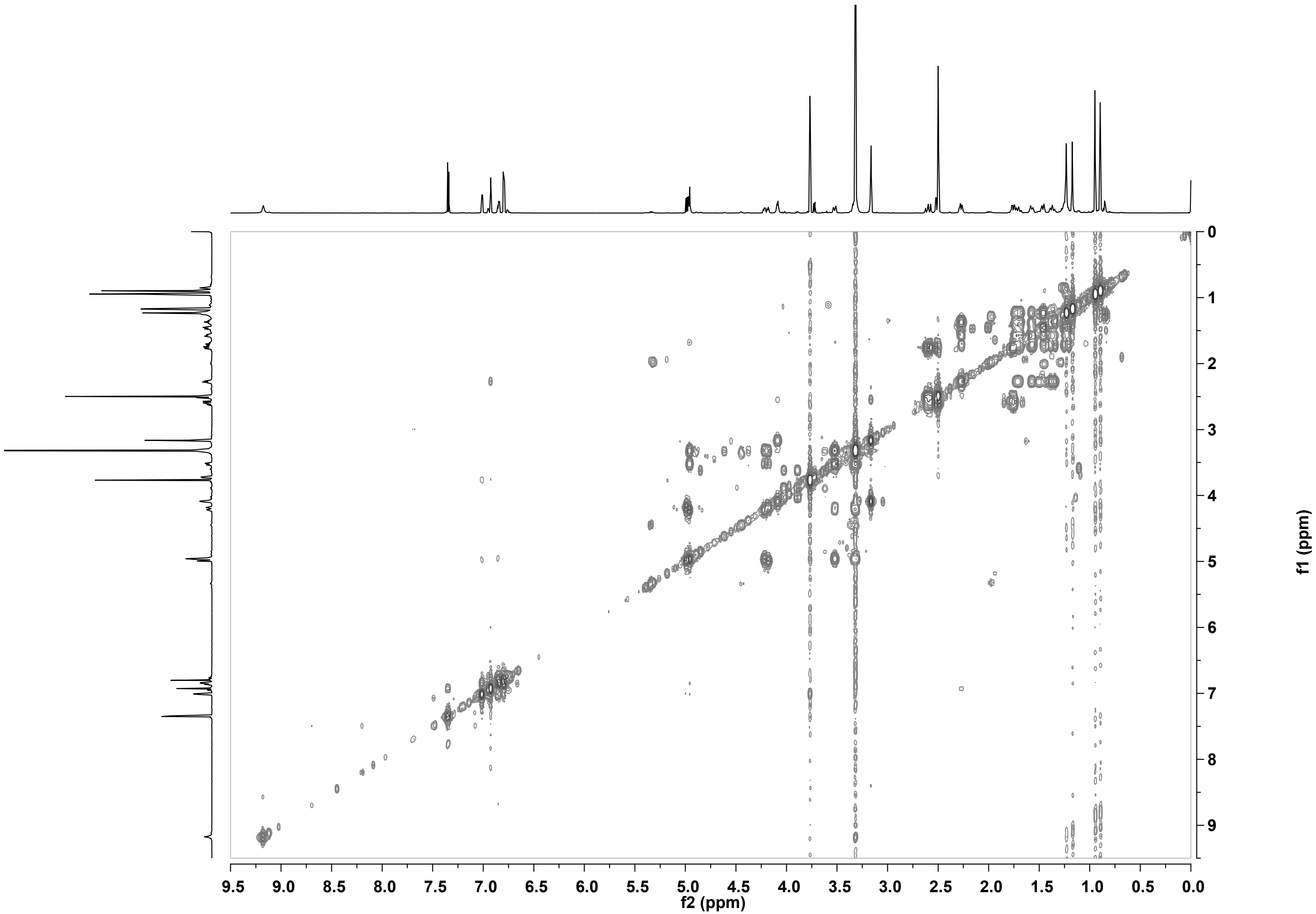

Figure S14. COSY (600 MHz, DMSO-d_6) spectrum of **3**

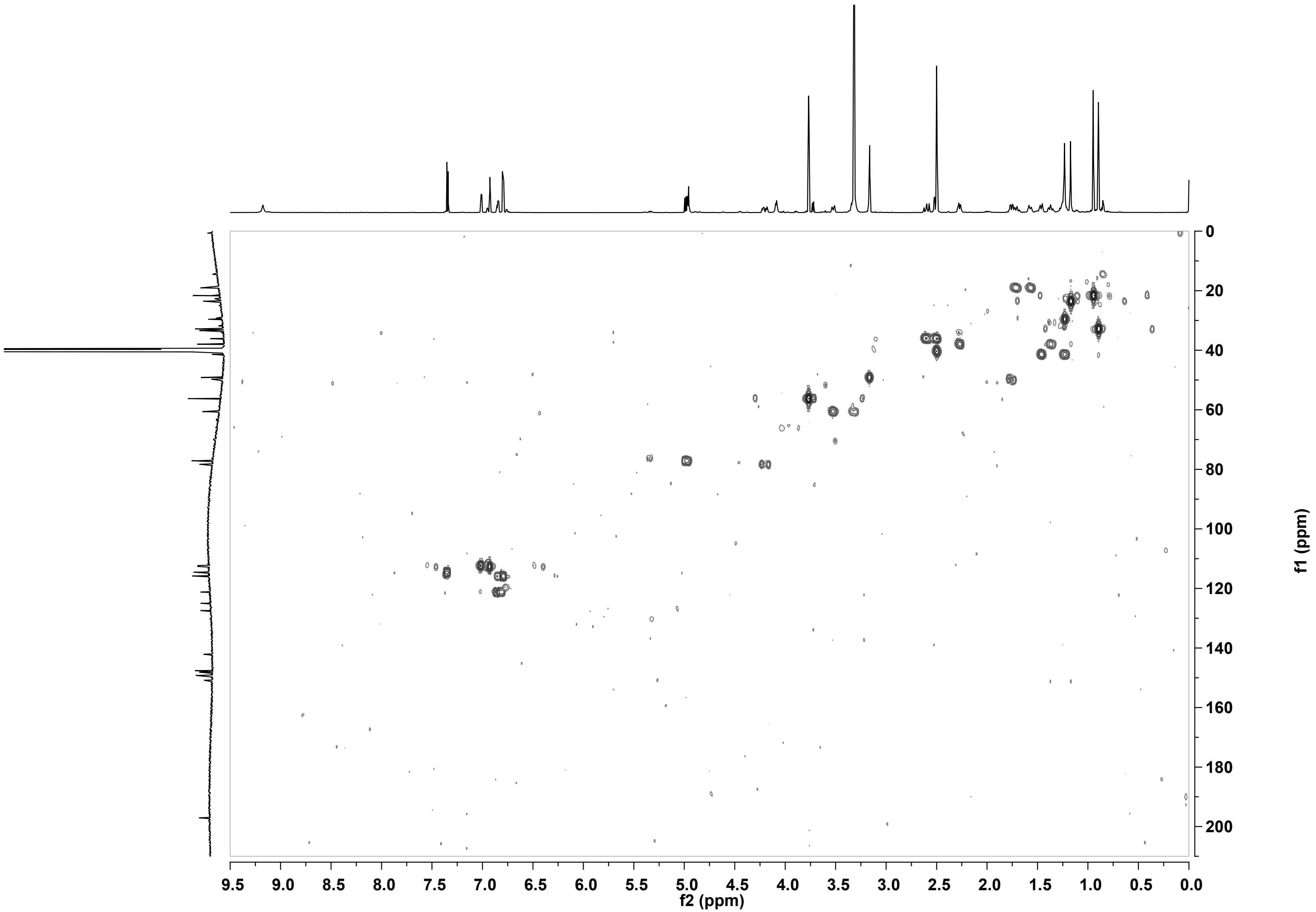

Figure S15. HMQC (600 MHz, DMSO-d_6) spectrum of **3**

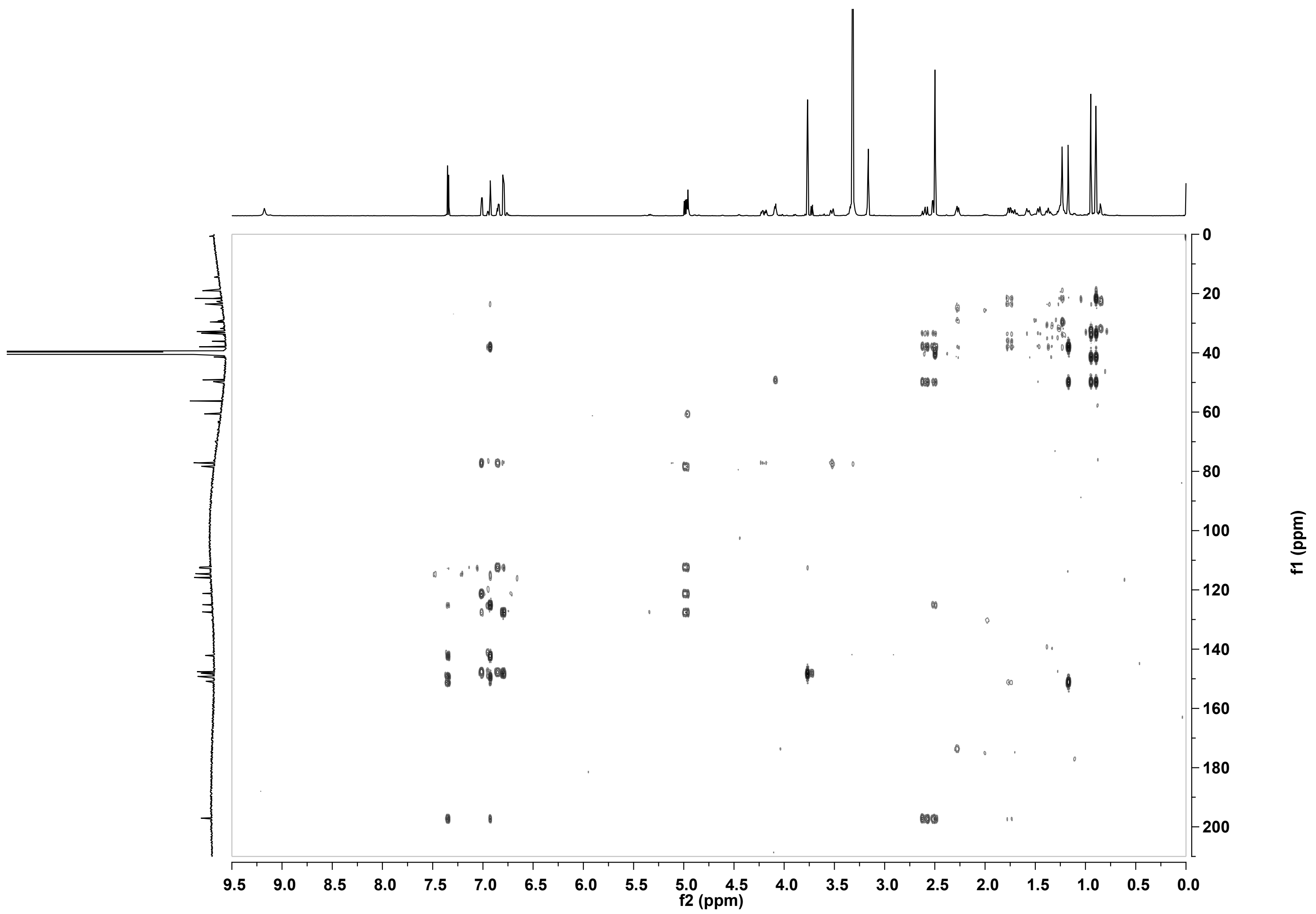

Figure S16. HMBC (600 MHz, DMSO-d_6) spectrum of **3**

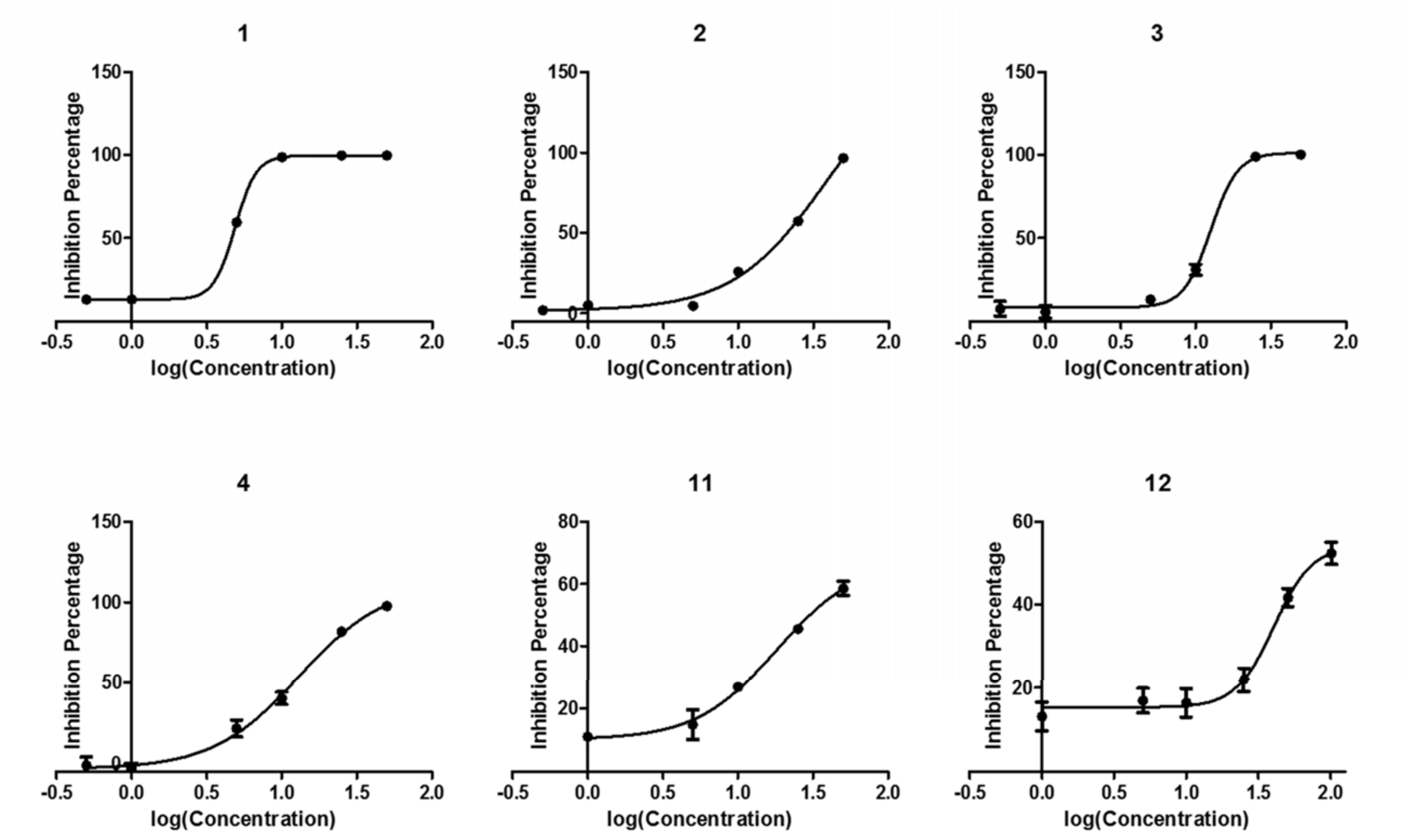

Figure S17. Inhibition percentage curves for the compounds **1**–**4**, **11**, and **12**

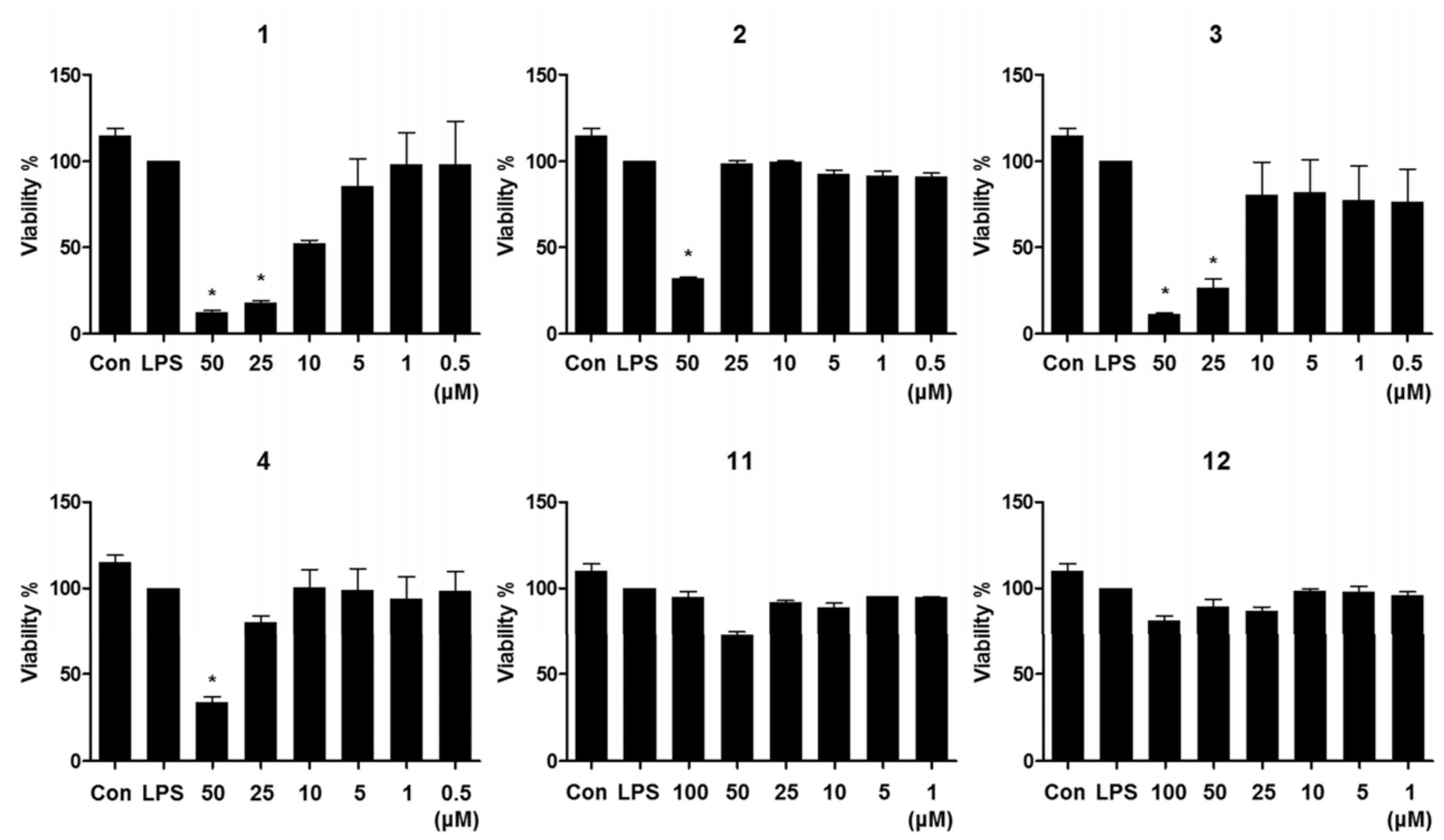

Figure S18. Cell viability for the compounds **1–4**, **11**, and **12**

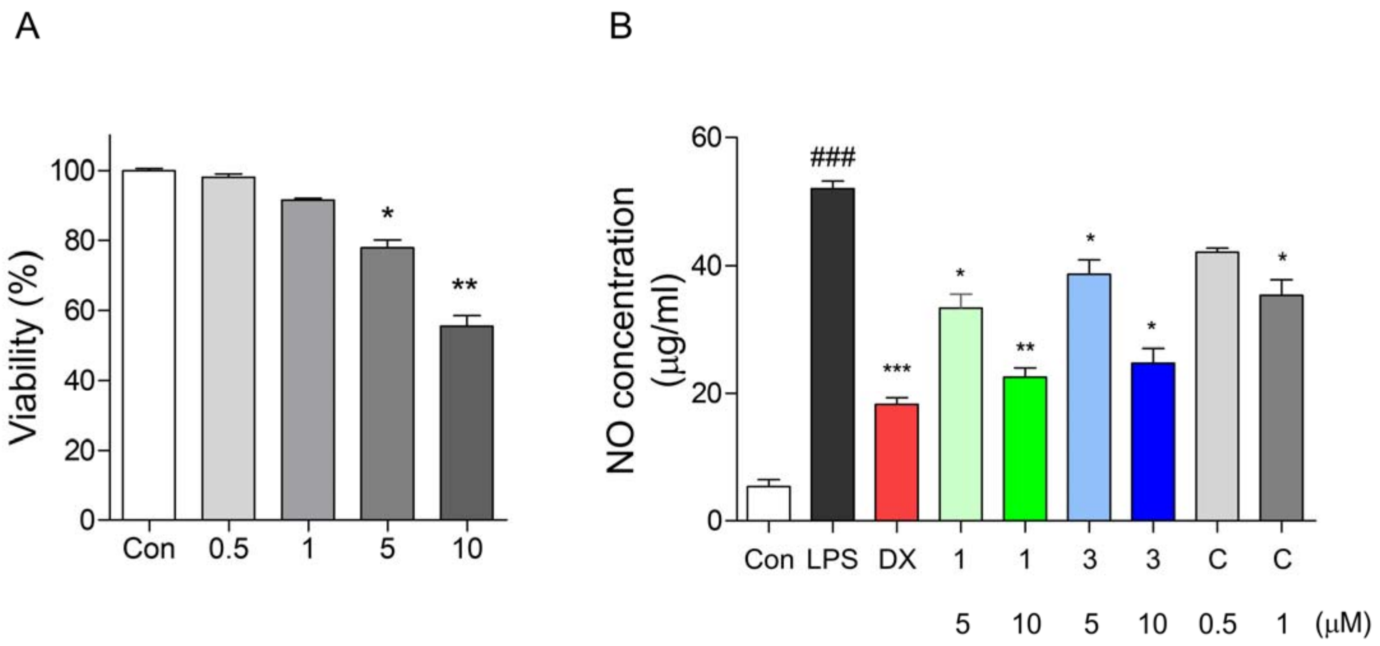

Figure S19. A comparison of Nitric oxide production between compound **1**, **3**, and celastrol in RAW 264.7 cells

(A) Cell viability was evaluated in 0.5 to 10 µM in celastrol. (B) NO concentration evaluated in compound 1, 3, and celastrol. Cells were pre-treated with each compound for 2 h and stimulated with LPS (1 µg/mL) for 16 h. Nitric oxide assay performed triplicate test, and results expressed as means ± SEM. An unpaired Student`s *t* test was used for statistical analysis. ###*P* < 0.001, **P* < 0.05,***P* < 0.01, and ****P* < 0.001 versus LPS. Con: control, LPS: lipopolysaccharide, Dx: dexamethasone, 1: compound 1, 3: compound 3, C: celastrol.
